# Biogenic silver/zinc oxide-carboxymethyl cellulose nanocomposites with enhanced photocatalytic and antimicrobial activities for environmental remediation

**DOI:** 10.1039/d6ra04414j

**Published:** 2026-09-15

**Authors:** Mohamed M. Hammouda, Khaled M. Elattar, Abdelrahman M. Abdelgawad, Dalia A. Elsherbiny, Abdulaziz A. Alanazi, Kamal Shalabi, Marwa M. Rashed

**Affiliations:** a Department of Chemistry, College of Science and Humanities in Al-Kharj, Prince Sattam Bin Abdulaziz University Al-Kharj 11942 Saudi Arabia m.elmettwaly@psau.edu.sa a.abdelgawad@psau.edu.sa d.elsherbiny@psau.edu.sa abdulaziz.alanazi@psau.edu.sa k.shalabi@psau.edu.sa; b Unit of Genetic Engineering and Biotechnology, Mansoura University El-Gomhoria Street Mansoura 35516 Egypt khaledelattar2@yahoo.com khaledelattar2@mans.edu.eg; c Toxicology Department, Mansoura Hospital, Faculty of Medicine, Mansoura University El-Gomhoria Street Mansoura 35516 Egypt marwarashed@mans.edu.eg

## Abstract

The development of a green methodology for the synthesis of silver/zinc/carboxymethyl cellulose (Ag/ZnO/CMC) nanocomposites using cinnamon extract as a reducing and stabilizing agent is reported herein. The innovation aspect of this research is the incorporation of biogenic Ag and Zn nanoparticles into the biodegradable CMC polymer matrix, resulting in the development of a versatile nanocomposite material with high antimicrobial and photocatalytic activities under visible light illumination. The nanocomposites were characterized by UV-Vis, FTIR, XRD, SEM, TEM, EDX, XPS, BET, and TPD techniques, confirming the mesoporous nature with Ag, AgCl, and ZnO crystals being uniformly distributed in the polymer matrix. The Ag/ZnO/CMC nanocomposite demonstrated exceptional antibacterial effects on Gram-positive and Gram-negative bacterial strains with an inhibition zone between 30.0 ± 0.8 and 36.0 ± 1.2 mm, which was higher than that achieved by the azithromycin antibiotic on various tested strains. The Ag/ZnO/CMC NC also showed broad-spectrum antibacterial activity, with MIC values of 3.125, 6.25, 6.25, and 12.5 µg mL^−1^ against *B. subtilis*, *K. pneumoniae*, *S. aureus*, and *S. typhimurium*, respectively. In addition, the nanocomposite attained 99.33 ± 1.00% photocatalytic degradation of the Crystal violet dye in 180 minutes under visible light illumination. From the kinetic study, an excellent match was found between the experimental data and the Langmuir–Hinshelwood kinetic model (*R*^2^ = 0.972). In addition, based on the analysis of thermodynamics, it was discovered that the reaction is spontaneous and endothermic. The nanocomposite remained stable with a degradation efficiency of over 91% after five rounds of recycling. The above results reveal that the nanocomposite is highly stable and recyclable. This research study is a sustainable platform for future eco-friendly multifunctional nanocomposites that will help in environmental remediation, wastewater purification, and antimicrobial protection.

## Abstract 2

Ag/ZnO/CMC showed strong antibacterial activity and visible-light degradation of Crystal violet, enhanced by CMC-stabilized dispersion and improved charge separation, ROS generation, and adsorption.

## Graphical abstract

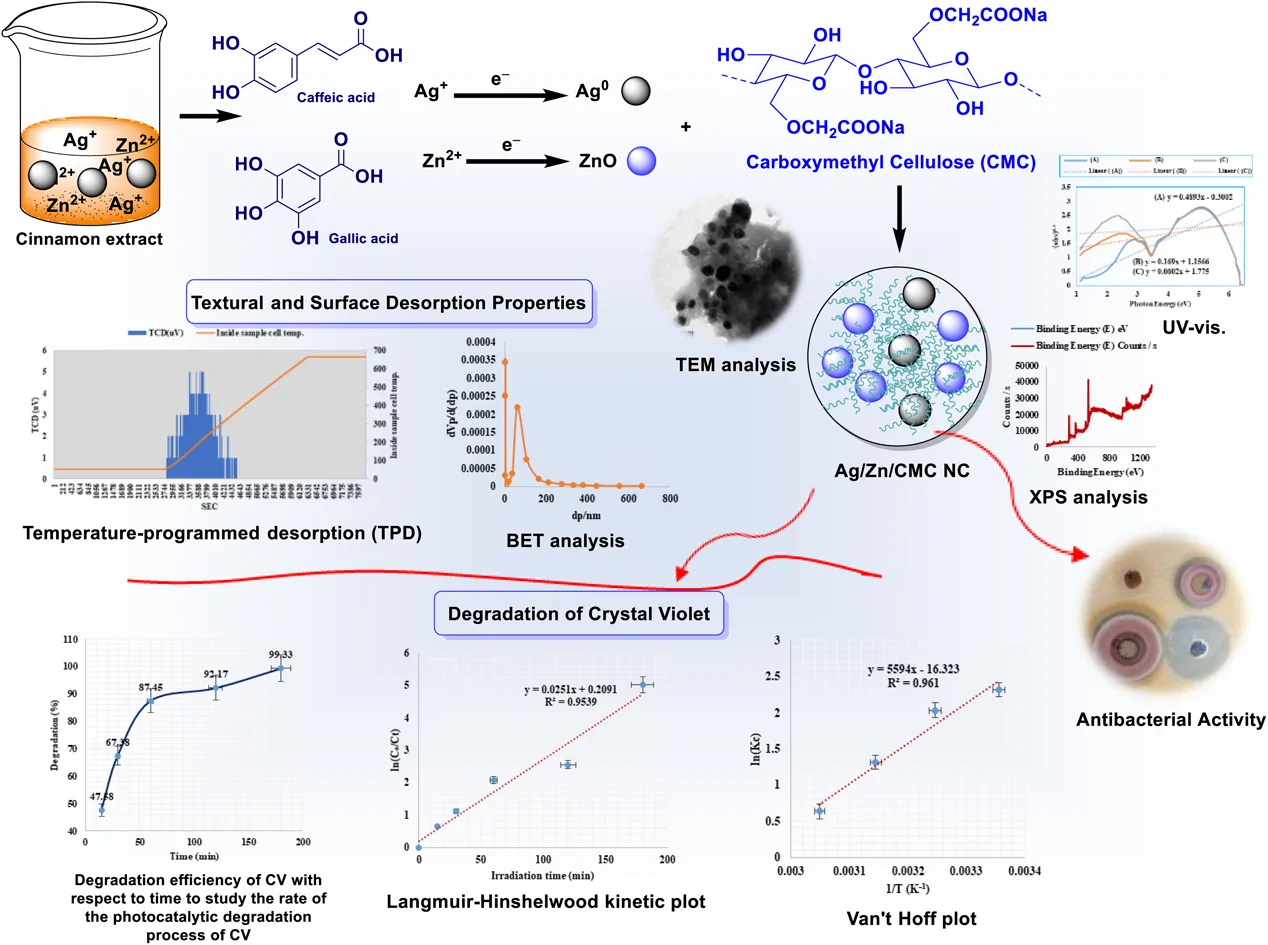

## 1 Introduction

Rapid industrial growth and urbanization have resulted in the generation of dangerous pollutants in the aquatic environment, particularly synthetic organic dyes produced by different sectors such as textiles, medicines, leather, cosmetics, and printing.^1^ Organic dyes are quite stable compounds with complex aromatic rings and are not subject to any biological processes. Due to these properties, organic dyes are considered dangerous to the environment and disturb the ecological balance in natural aquatic environments. Apart from the reduction in light transmittance and dissolved oxygen concentration in water bodies, most organic dyes and their metabolites are toxic, mutagenic, and carcinogenic to aquatic species and human beings.^1^ Crystal violet is one of the most dangerous and difficult-to-remove classes of pollutants due to its stability, resistance, and toxicity. Crystal violet can cause dermatitis, pulmonary infections, genotoxicity, and even cancer when exposed to for long periods of time. All these factors make Crystal violet dye a great threat to the environment and human health.^2^

There are traditional water treatment methods that have long been used for the removal of dyes from wastewater, including coagulation, adsorption, filtration, and biological treatment. Nevertheless, these techniques have several disadvantages, like incomplete dye breakdown, expensive maintenance, production of sludge, creation of secondary contamination, and inefficiency with chemically stable compounds.^3^ Therefore, there is an increasing need for novel and eco-friendly methodologies that would allow for the full degradation of toxic dyes. Advanced oxidation processes (AOPs) can be considered extremely promising solutions due to their capability of producing reactive oxygen species (ROS) such as hydroxyl radicals (˙OH) and superoxide radicals (O_2_˙^−^) that can decompose and destroy recalcitrant organic contaminants.^4^

Out of the many available approaches, semiconductor-based photocatalysis has gained significant interest as a green and energy-saving process for wastewater purification.^5^ Photocatalysis can harness the energy from sunlight or visible light to create electron–hole pairs, thus enabling the oxidation processes of the organic pollutants. Moreover, hybrid adsorption–photocatalysis processes have shown improved pollutant removal capability due to the synergy of the adsorptive and photocatalytic activities.^6^ The development of photocatalytic processes for water treatment has been greatly boosted by the development of nano-sized photocatalysts with a large surface area, adjustable optical behavior, fast charge transfer, and high catalytic activity.^7^

Nanomaterials prepared from metals and metal oxides, especially those containing silver and zinc, have been known to display excellent photocatalytic properties and antimicrobial performance under the irradiation of visible light.^8,9^ The introduction of noble metals and multi-functionality in the semiconductors could assist in achieving the separation, increase the ability of absorbing visible light, and prevent the electron–hole recombination; hence, improving the photocatalytic efficiency. Nevertheless, the traditional approach of producing these nanomaterials often needs toxic chemicals, harmful solvents, and heat energy.

To overcome these challenges, several studies have taken place on the approaches of green synthesis for developing environmentally friendly and sustainable nanomaterials.^10,11^ The plant-assisted method is based on the utilization of natural phytochemicals, including phenols, flavonoids, tannins, alkaloids, and terpenoids, which can reduce, stabilize, and protect nanoparticles from agglomeration.^12^ Not only does this approach help minimize the toxicity associated with other synthetic methods, but it also increases the biological and surface reactivity of the produced nanomaterials. Among numerous medicinal plants, the *Cinnamomum verum* (cinnamon) has shown great potential since its extracts include abundant phenolic compounds, cinnamaldehyde, and flavonoids, which can be used for nanoparticle synthesis and stabilization.^13,14^

In terms of enhancing the stability and dispersity of nanomaterials, the formulation of polymer matrices is very significant. Carboxymethyl cellulose (CMC) is a bio-degradable hydrophilic biopolymer that has received much attention as a functional polymer matrix material for developing nanocomposites owing to its film-forming ability, high solubility in water, and abundance of hydroxyl or carboxyl groups.^15,16^ The addition of metal nanoparticles to the CMC matrix will not only improve their dispersion but also inhibit their agglomeration and promote interactions at the interface. This results in better physical/chemical and catalytic performance. In addition, the synergism between the metallic nanostructure and polymeric support will greatly improve catalytic efficiency, charge transfer dynamics, and adsorption capacity of nanocomposites.^17^

Photocatalytic degradation of organic dyes using nanocomposites made up of semiconductors typically occurs *via* the production of ROS in light-activated reactions. After excitation with photons, electrons move from the valence band to the conduction band, creating electron–hole pairs that later interact with oxygen and water to form highly reactive oxidative agents that facilitate the degradation of dye molecules.^18,19^ Apart from being effective in photocatalytic activity, metal-based nanocomposites have been shown to exhibit strong antibacterial activity because of their ability to produce ROS, disrupt membranes, oxidize proteins, and cause DNA damage.^20^ Multifunctional nanocomposites with dual functionality are therefore highly desirable for wastewater treatment purposes.

Although remarkable advancements have been made in green nanotechnology, only a few works have been reported in the literature concerning the incorporation of plant-mediated silver/zinc-based nanoparticles into biodegradable CMC matrices for photocatalytic degradation and antibacterial activity. In addition, no detailed understanding of the interrelation between green synthesis, stabilization by polymers, visible light photocatalysis, and antibacterial efficacy has been provided. This study was designed to develop a multifunctional metal/polymer nanocomposite *via* green synthesis using *Cinnamomum verum* extract as a reducing and stabilizing agent in a carboxymethyl cellulose matrix. The synthesized nanocomposite material was analyzed using various physicochemical methods and studied for its visible light-induced degradation efficiency towards Crystal violet dye, as well as its antibacterial properties. The present work also examines the effect of the combination of biologically produced phytochemicals, metal nanoparticles, and polymeric matrix on the enhancement of catalytic and biological activity of the nanocomposites.

## 2 Materials and methods

### 2.1. Reagents and chemicals

All chemicals and reagents used were of analytical grade without further purification. Silver nitrate (AgNO_3_), zinc acetate dihydrate [Zn(CH_3_COO)_2_·2H_2_O], and carboxymethyl cellulose (CMC) were used for nanocomposite synthesis, while Crystal violet (CV) dye was employed for photocatalytic studies. HCl and NaOH were used for pH adjustment, CaCl_2_ for ionic strength studies, and ethanol/distilled water for purification procedures. Nutrient media were used for antibacterial assays. Fresh bark of *Cinnamomum verum* was utilized for the preparation of the aqueous extract as a natural reducing and stabilizing agent (Section S1).

### 2.2. Instruments

UV-Vis spectroscopy was performed using a Spekol 11 spectrophotometer (Analytik Jena AG, Germany). FTIR spectra were recorded using a Bruker spectrometer, while XRD analysis was conducted using a PANalytical Philips diffractometer. Surface morphology and elemental composition were analyzed using SEM-EDX, and TEM analysis was used to investigate nanoparticle morphology and size distribution. Sonication was carried out using an Elma Schmidbauer sonicator, and centrifugation was performed using a Beckman Coulter Allegra X-15R centrifuge. Surface area analysis was conducted using a BELSORP MINI X analyzer, while CO_2_-TPD and NH_3_-TPD analyses were applied to evaluate surface acidic/basic properties and catalytic active sites of the synthesized nanocomposites (Section S1).

### 2.3. Preparation of aqueous cinnamon extract

The fresh bark of *Cinnamomum verum* was gathered, cleaned with distilled water to eliminate any foreign matter such as dirt and other impurities, and then dried in the open air at 25 ± 2 °C for five to seven days until the substance acquired constant weight. The dried product was ground into a fine powder using a sterilized electric grinder. For the extraction procedure, 10 g of powdered cinnamon was added to 100 mL of distilled water in a 250 mL conical flask and heated to 70 °C for 30 minutes while continuously stirred by a magnet (600 rpm). The solution was cooled to room temperature and then filtered through a muslin cloth and finally a Whatman No. 1 filter paper to separate any large plant fragments. The filtrate was further subjected to centrifugation at 6000 rpm for ten minutes to yield a clear aqueous extract free of any suspended impurities. The final aqueous extract of cinnamon was stored in sterilized and amber glass bottles at 4 °C, to be utilized within one week for retention of the bioactivity of its phytocompounds. This extract acted as a reducing, stabilizing, and capping agent owing to its high concentration of polyphenolic, flavonoid, and cinnamaldehyde compounds.^21^

### 2.4. Green synthesis of nanocomposites

#### 2.4.1. Green synthesis of Ag/ZnO nanocomposite

Ag/ZnO NC synthesis was done using a green approach referred to as the co-reduction method,^22^ where an aqueous cinnamon extract functions as both a reducing and stabilizing agent. Usually, 50 mL of AgNO_3_ (silver nitrate) with a concentration of 1 mM is mixed with 50 mL of zinc acetate dihydrate [Zn(CH_3_COO)_2_·2H_2_O] with a concentration of 1 mM by stirring magnetically with a speed of 600 rpm at a room temperature of 25 ± 2 °C. An equimolar Ag : Zn precursor ratio (1 : 1, mol mol^−1^) was selected to provide a balanced incorporation of the Ag and ZnO phases and to facilitate their anticipated synergistic interaction during photocatalysis and antibacterial activity. This Ag/ZnO NC loading was selected as the fixed formulation investigated throughout the present study and was not subjected to systematic loading optimization. Afterward, 20 mL of freshly extracted cinnamon was gradually added to the mixture at 1 mL per minute, then the mixture was heated to 70 °C for 2 hours. Formation of Ag/ZnO nanocomposite was confirmed by the color change from pale yellow to brownish-gray suspension. This process was carried out three to four times, where distilled water was followed by ethanol washing in order to remove any remaining reactant ions, plant biomolecules, and other soluble contaminants. The washing process was followed by centrifuging at a speed of 10 000 rpm for 10 minutes. The resulting solid nanocomposite material was dried at 70 °C in a hot air oven for 12 hours. It should be noted that drying continued until the stable weight was achieved. The obtained dried Ag/ZnO nanocomposite material was finely ground using a mortar and pestle made of agate. The dried Ag/ZnO nanocomposite was stored in sterile glass ampules under a layer of silica gel to avoid moisture sorption and nanocomposite clustering.

#### 2.4.2. Synthesis of Ag/ZnO/CMC nanocomposite

The Ag/ZnO/CMC NC was prepared by embedding the pre-synthesized Ag/ZnO nanocomposite, obtained using an equimolar Ag : Zn precursor ratio (1 : 1, mol mol^−1^), into a carboxymethyl cellulose (CMC) polymer matrix.^23^ The selected Ag/ZnO NC-to-CMC ratio was maintained throughout the study to ensure a consistent and reproducible formulation for subsequent structural, photocatalytic, and antibacterial evaluations. Initially, 1.0 g of CMC was dissolved in 100 mL of distilled water under continuous stirring at 60 °C (700 rpm) until a clear, homogeneous, and viscous solution was obtained. Separately, 0.50 g of dried Ag/ZnO NC was dispersed in 20 mL of distilled water and ultrasonicated for 30 min to ensure uniform dispersion. Thus, the Ag/ZnO NC-to-CMC mass ratio was fixed at 1 : 2 (w/w). The dispersed Ag/ZnO NC suspension was then slowly added into the CMC solution under vigorous stirring at 700 rpm, followed by continued stirring at 70 °C for 3 h to ensure strong interaction between the hydroxyl/carboxyl groups of CMC and the nanoparticle surface. The mixture was further sonicated for 20 min to improve homogeneity. The resulting composite hydrogel-like suspension was centrifuged at 8000 rpm for 15 min to collect the solid nanocomposite phase. The precipitated material was washed thoroughly with distilled water (3 cycles) to remove unbound polymer chains and loosely attached nanoparticles. The purified Ag/ZnO/CMC nanocomposite was then freeze-dried (lyophilized) at −50 °C under 0.1 mbar vacuum for 24 h to obtain a highly porous solid structure. Alternatively, for conventional drying, the material was oven-dried at 60 °C for 24 h until constant weight was achieved, depending on the availability of equipment. The dried nanocomposite was gently ground into a fine powder using an agate mortar and pestle. The final Ag/ZnO/CMC nanocomposite powder was stored in airtight amber glass containers and kept in a desiccator at room temperature (25 ± 2 °C). To preserve structural integrity and prevent moisture-induced aggregation or polymer swelling, the samples were protected from humidity and direct light exposure. The stored nanocomposite remained stable and was used for all photocatalytic and adsorption experiments without further modification.

### 2.5. Antibacterial assessment

#### 2.5.1. Agar well diffusion assay

The antibacterial efficacy of *Cinnamomum verum* extract and the synthesized nanocomposites was determined by employing the well diffusion assay technique against some selected strains of bacteria.^28,29^ For this, nutrient agar (NA) media were made by dissolving the requisite amount of nutrient agar powder in distilled water, followed by autoclaving at 121 °C for 15 minutes under 15 psi pressure. The autoclaved media were then transferred to sterile Petri plates after solidification at room temperature in a laminar airflow environment. Bacterial cultures were grown in fresh nutrient broth media for 18–24 hours at 37 °C in order to get them in their exponential growth phase. After this, the inoculum was adjusted to the 0.5 McFarland standards (1 × 10^8^ CFU mL^−1^).

The sterile cotton swab was used to streak the bacterial suspension evenly across the agar plate surfaces to provide an even growth of bacteria on the plates. The wells of about 6 mm in diameter were drilled aseptically into the agar by using a sterile cork borer. The Ag/ZnO/CMC NC (100 µg mL^−1^) was prepared in sterile distilled water. Each well was filled aseptically with 50–100 µL of the prepared suspension of nanocomposite under laminar air flow conditions. Sterile distilled water and antibiotic solution served as negative and positive controls, respectively. After that, the agar plates were incubated for 1 hour at ambient temperature to allow pre-diffusion of the sample into the agar before incubation at 37 °C for 24 hours. The antibacterial activity was determined by measuring the diameter of the inhibition zones (incorporating well diameter) in mm using a calibrated scale or digital micrometer. All tests were conducted in triplicate, and the results were presented as mean ± SD. Antibacterial efficiency was judged from the inhibition zone diameter, whereby greater inhibition zones reflected greater antibacterial activity due to better interaction with the bacterial cell walls as well as the release of the bioactive metal ions.

#### 2.5.2. Broth microdilution assay for MIC determination

The minimum inhibitory concentration (MIC) of the Ag/ZnO/CMC NC against the tested bacterial strains was determined using a broth microdilution assay based on OD_600_ measurements, with the MIC defined as the lowest NC concentration showing complete inhibition of bacterial growth. The detailed experimental procedure is provided in Section S1 of the SI.

### 2.6. Photocatalytic degradation of Crystal violet using ag/ZnO/CMC nanocomposite

#### 2.6.1. Preparation of Crystal violet dye solution

A stock solution of Crystal violet (CV) dye (1000 mg L^−1^) was prepared by dissolving an accurately weighed amount of Crystal violet powder in distilled water. Working solutions of the desired concentration (50 mg L^−1^) were freshly prepared by appropriate dilution of the stock solution before each experiment.

#### 2.6.2. Photocatalytic degradation experiment

All photodegradation experiments were performed on the prepared Ag/ZnO/CMC nanocomposite (NC) *via* visible light illumination. For example, in one such experiment, 50 mL of Crystal violet was added to a 100 mL conical flask, and after that, a certain quantity of the Ag/ZnO/CMC NC (0.10–1.00 mg mL^−1^) was added. The reaction mixture was stirred magnetically for 30 minutes in the dark until the dye molecules and the nanocomposite reached the adsorption–desorption equilibrium state. Subsequently, the mixture was illuminated by means of a 300 W xenon lamp that served as the visible light source while still stirring magnetically. Samples were then collected periodically within different time intervals (15–180 min) and subsequently subjected to centrifugation at 10 000 rpm for 10 minutes.

#### 2.6.3. Effect of operational parameters

The effects of various operating conditions on the degradation performance of the photocatalytic process were studied.^24^ Dye solution was made acidic or alkaline in the pH range 2–10 with the use of 0.1 M HCl or 0.1 M NaOH. Irradiation time effect ranged from 15 to 180 minutes, and reaction temperature changed to 25–55 °C. Furthermore, various dosages of Ag/ZnO/CMC NC (from 0.10 to 1.00 mg mL^−1^) were tested. Finally, ionic strength was changed with various doses of CaCl_2_ solution (from 0 to 2500 µL).

#### 2.6.4. UV-visible spectrophotometric analysis

Quantitative determination of the photocatalytic degradation activity of Crystal violet dye *via* the synthesized Ag/ZnO/CMC nanocomposite (NC) was performed through UV-Visible spectrophotometry.^25^ After certain exposure periods, about 3 mL samples were withdrawn from the reaction mixture and then centrifuged at a speed of 10 000 rpm for 10 minutes to fully separate the nanocomposite particles from the reaction mixture to avoid further photocatalytic reaction during the measurement process. The resulting supernatant was analyzed using a UV-Vis spectrophotometer, ranging from wavelengths of 200–800 nm.

The leftover concentration of Crystal violet dye was obtained based on the maximum absorption (*λ*_max_ = 590 nm). This is since changes in the absorbance of the solution are proportional to the degradation of the dye molecules under visible light illumination. Degradation efficiency (%) was determined using the equation below:Degradation efficiency (%) = (*C*_0_ − *C*_*t*_)/*C*_0_ × 100where *C*_0_ is the initial concentration of Crystal violet dye before visible light irradiation, and *C*_*t*_ is the residual dye concentration remaining in the solution after irradiation time *t*.

Calibration curves based on the Crystal violet standards were prepared to accurately determine the concentration, showing high linear correlation within the chosen concentration range (*R*^2^ > 0.99). Besides efficiency testing, the time-dependent changes of the absorption spectra were also studied to investigate the decomposition of the chromophore structure of Crystal violet molecules. The absence of extra absorption bands alongside a decrease in the intensity of the absorption peak at 590 nm, indicated the effective degradation of the CV chromophore by the Ag/ZnO/CMC NC. However, complete mineralization cannot be confirmed solely from UV-Vis measurements and would require complementary analyses such as TOC or LC-MS. The experiments were carried out three times, and all the results were presented as mean ± SD values.

#### 2.6.5. Reusability test of Ag/ZnO/CMC nanocomposite

The reusability behavior of the fabricated Ag/ZnO/CMC nanocomposite (NC) was assessed *via* repetitive degradation experiments of Crystal violet (CV) dye under the optimal reaction conditions.^26^ In brief, 50 mL of Crystal violet solution (50 mg L^−1^) was degraded by using an optimized quantity of Ag/ZnO/CMC NC in the presence of visible light irradiation for 180 minutes. After each cycle, the nanocomposite was isolated from the reaction mixture *via* centrifugation at 10 000 rpm for 10 minutes. The recovered nanocomposite was repeatedly washed several times with distilled water, and subsequently, ethanol was utilized to eliminate any adsorbed dye molecules and reaction products from the surface of the catalyst. The washed nanocomposite was dried at 60 °C in a hot air oven for 12 hours before its reuse in the following degradation cycle under the same reaction parameters. The percentage degradation (%) was determined after each cycle to assess the catalytic stability and recycling ability of Ag/ZnO/CMC NC during multiple utilization. Five successive cycles were performed, and all experiments were carried out in triplicate with results expressed as mean ± standard deviation (SD).

#### 2.6.6. Comparative photocatalytic experiments and blank control

In order to confirm the synergistic role of the Ag/ZnO/CMC nanocomposite in photocatalysis and separate the effect of its components, degradation studies were carried out using the same experimental conditions. CV solution (50 mL, 50 mg L^−1^) was studied in the presence of CMC, Ag/CMC, ZnO/CMC, physical mixture of Ag and ZnO/CMC, and the synthesized Ag/ZnO/CMC NC. The mass of the catalyst, initial CV concentration, pH, light exposure time, temperature, stirring conditions, and the visible light source were the same in all the experiments. In the case of the physical mixture, the physical mixture of Ag/CMC and ZnO/CMC was used in the same ratio as in the synthesis of the Ag/ZnO/CMC NC.

Also, a blank test was carried out with 50 mL CV solution (10 mg L^−1^) in the absence of catalyst under identical conditions of visible light irradiation. In the blank test, there was no use of photocatalyst, which was kept at pH 10 for 180 min at 25 °C. It was conducted in order to determine the amount of degradation due to the direct photolysis of CV. All experiments were performed in triplicate, and the results are expressed as mean ± SD.

#### 2.6.7. Reversibility and water matrix stability test

Photocatalytic activity and operational stability of the Ag/ZnO/CMC NC were further tested using various water matrices such as distilled water, tap water, and seawater.^27^ Crystal violet dye solutions (50 mg L^−1^) were first prepared using each water matrix, and then the optimized amount of Ag/ZnO/CMC NC was added. The dye removal tests were conducted under visible light exposure, and the optimum parameters of pH, irradiation time, and catalyst dosage were the same for all types of water matrices. Before the irradiation, the samples were stirred in the dark for 30 minutes to attain the adsorption–desorption equilibrium state. Then, the dye concentrations were measured spectroscopically at 590 nm using the UV-Vis spectrometer. Degradation efficiency in each water medium was compared to determine the influence of various dissolved salts, minerals, and other impurities in water on the photocatalytic behavior of the nanocomposites. The photocatalytic efficiency maintained in various water matrices confirmed the environmental feasibility, reversibility, and operational stability of the Ag/ZnO/CMC NC for dye degradation.

#### 2.6.8. Determination of Ag^+^ and Zn^2+^ leaching

The release of Ag^+^ and Zn^2+^ from the Ag/ZnO/CMC nanocomposite (NC) was studied using an atomic absorption spectrophotometer produced by Buck Scientific (USA). The Ag/ZnO/CMC NC was dissolved in CV solution under various catalytic concentrations, following the photocatalytic conditions used in the degradation experiments. After the completion of irradiation process, the samples were centrifuged to separate the photocatalyst from the mixture. The supernatants were collected after filtration and analyzed using the AAS method. To calculate the amount of Ag and Zn in the Ag/ZnO/CMC NC, known masses of dried NC were dissolved in sulfuric acid. Heating was carried out slowly and in a controlled manner until all the samples underwent digestion to yield a well-defined solution. Upon cooling to room temperature, the digest samples were transferred quantitatively into volumetric flasks and diluted using ultrapure water up to a defined volume. Blanks, which included only sulfuric acid without the nanocomposite material in the same volume, were prepared and underwent the exact digestion process. Matrix controls were also prepared from CMC alone and digested through the exact procedure.

Ag and Zn levels in the digested catalysts, supernatant of the photocatalysis reaction mixture, reagent blanks, and CMC controls were measured by AAS at the required wavelength with calibration curves made from standard solutions of Ag and Zn. The calibration standard solutions were prepared in a sulfuric acid solution similar to that of the sample solutions. The blank signal was deducted from the signal of the respective sample solution. All analyses were done in three replicates and results reported as mean ± SD. The percentage of metal leaching was calculated according to:Leaching (%)= (*C* leached × *V*)/*m* total metal × 100where *C* leached is the concentration of Ag or Zn in the supernatant after the photocatalysis process (mg L^−1^), *V* is the volume of the reaction mixture (L), and *m* total metal is the total weight of Ag or Zn in the Ag/ZnO/CMC NC.

#### 2.6.9. Radical-scavenging experiments

In order to determine the major reactive species responsible for photocatalytic degradation of Crystal violet (CV), a series of selective radical scavenging tests was carried out under the optimized conditions for photocatalysis. IPA, BQ, EDTA-2Na, and NaN_3_ were used as scavengers for ˙OH radicals, O_2_˙^−^ radicals, h^+^ photogenerated holes, and singlet oxygen (^1^O_2_), respectively. In brief, Ag/ZnO/CMC NC was added into the CV solution under the optimized catalyst loading and initial CV concentration. Before irradiation, the suspension was magnetically stirred in the dark until adsorption–desorption equilibrium was attained. Afterward, the proper scavenger was introduced into the reaction mixture at the respective concentrations of 10 mM for IPA and 1 mM for BQ, EDTA-2Na, and NaN_3_. Thereafter, the suspension was irradiated using similar visible light illumination as in the photodegradation experiments. The samples were withdrawn at the specified reaction time and the catalyst was separated from the suspension *via* centrifugation after which the CV concentration remaining in the suspension was measured through UV-Vis spectrophotometer analysis at its maximum absorption wavelength. Control experiments without the presence of scavengers were also performed under the same conditions. All experiments were carried out in triplicate and results expressed as mean ± SD. The inhibitory effect of the scavengers was quantitatively determined as:Inhibition (%) = (*D*_0_ − *D*_s_)/*D*_0_ ×100where *D*_0_ is the percentage of CV degradation in the absence of scavenger and *D*_s_ is the percentage of CV degradation in the presence of scavenger. The significant reduction in CV degradation upon the addition of a particular scavenger is used as an indication of the important role played by the corresponding reactive species in the process of degradation.

### 2.7. Statistical analysis

All experiments were conducted in triplicate (*n* = 3), and the obtained results were expressed as mean ± standard deviation (SD). Statistical analysis was performed using GraphPad Prism software (version 9.0, GraphPad Software, San Diego, CA, USA) and Microsoft Excel 365. Differences between experimental groups were evaluated using one-way analysis of variance (ANOVA) followed by Tukey's *post hoc* multiple comparison test to determine statistically significant differences among treatment conditions. The probability value of *p* < 0.05 was considered statistically significant. Linear regression analysis was applied for kinetic, adsorption isotherm, and thermodynamic modeling, and the goodness of fit of each model was evaluated using the correlation coefficient (*R*^2^). Error bars in graphical representations correspond to the standard deviation of three independent experiments.

## 3 Results and discussion

### 3.1. Mechanism of the formation of nanocomposites

**Fig. 1:**
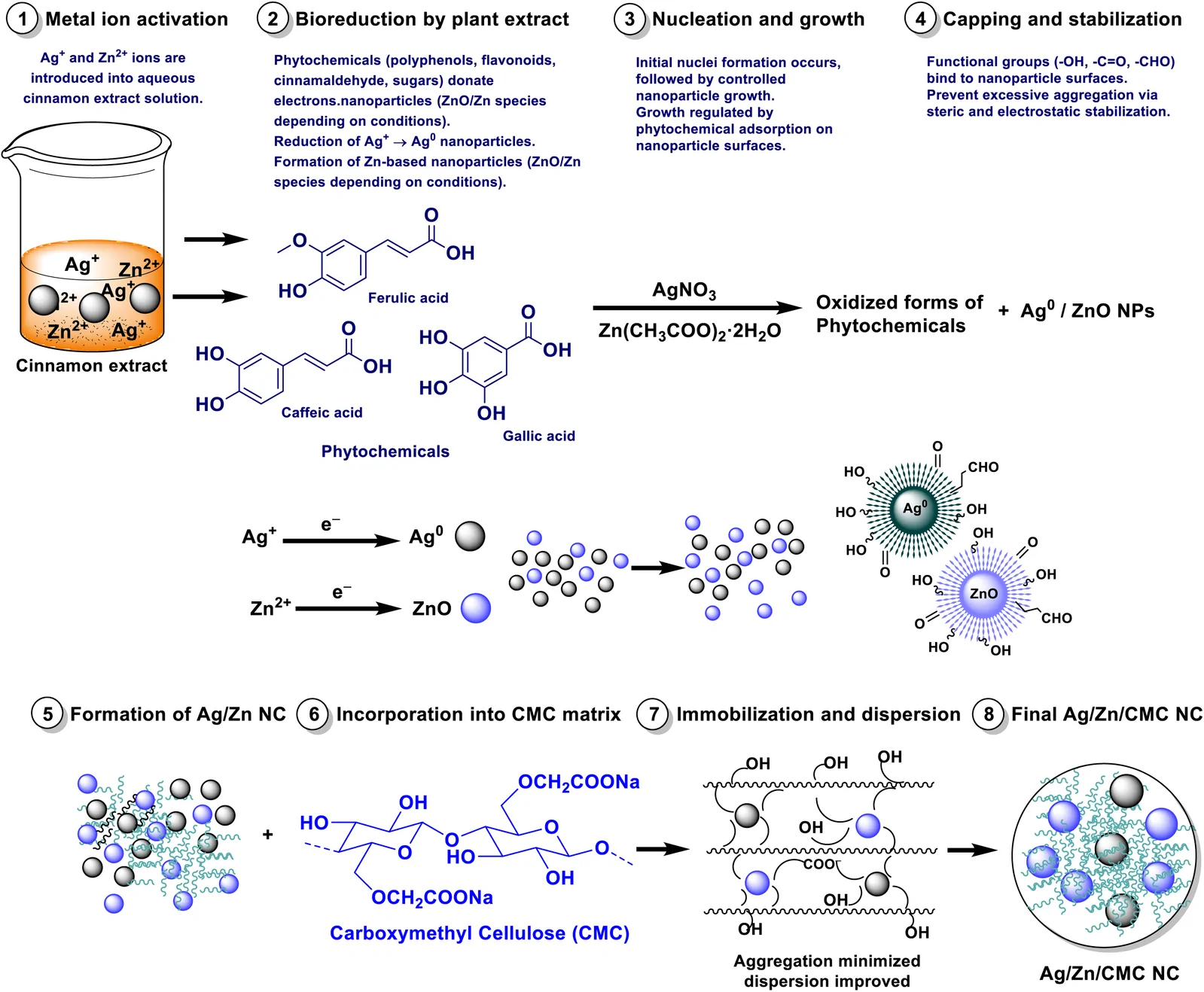
Proposed green synthesis mechanism of Ag/ZnO and Ag/ZnO/CMC nanocomposites using cinnamon extract. Phytochemicals present in cinnamon extract, including polyphenols, flavonoids, cinnamaldehyde, and sugars, act as reducing and stabilizing/capping agents during the reduction of Ag^+^ and Zn^2+^ ions and subsequent formation of Ag/ZnO nanoparticles. The nanoparticles undergo nucleation and growth followed by surface stabilization through interactions with phytochemical functional groups. Subsequent incorporation into the CMC matrix is proposed to occur through hydrogen bonding and coordination interactions, yielding stabilized Ag/ZnO/CMC nanocomposites.

The green formation of the Ag/ZnO and Ag/ZnO/CMC nanocomposites is proposed to involve a sequence of phytochemical-mediated metal-ion transformation, nucleation, growth, surface stabilization, and incorporation of the inorganic nanophases within the CMC matrix (Fig. 1). In the initial stage, bioactive constituents present in the aqueous cinnamon extract, particularly polyphenols, flavonoids, cinnamaldehyde, and reducing sugars, may interact with Ag^+^ and Zn^2+^ precursor ions, facilitating the formation of metallic Ag^0^ and ZnO nanophases, respectively, while the associated phytochemical functional groups may contribute to their nucleation and surface stabilization.

Cinnamon extract is particularly attractive for this phytogenic synthesis because of its chemically diverse composition and abundance of multifunctional oxygen-containing compounds. Cinnamaldehyde, phenolic compounds, flavonoids, and reducing sugars provide different functional groups capable of participating in metal-ion coordination, surface adsorption, and redox processes. This multifunctional phytochemical environment can simultaneously contribute to the formation and surface stabilization of the developing inorganic nanophases, reducing the need for conventional synthetic reducing and capping agents. Moreover, phytochemical residues may remain associated with the nanoparticle surfaces after synthesis, providing additional surface functionality that may contribute to the biological and redox properties of the resulting material.

Following nucleation, the growth of the Ag/ZnO nanophases is proposed to occur through progressive deposition and interaction of the developing inorganic species with functional groups originating from the cinnamon extract. In particular, hydroxyl, carbonyl, and aldehyde groups may interact with the particle surfaces, thereby influencing nucleation, growth, and surface capping. These interactions can provide a degree of steric protection and help limit uncontrolled particle growth. The phytochemical constituents should therefore be considered surface-associated capping/stabilizing components rather than impurities in the green-synthesized nanocomposite.

The subsequent incorporation of CMC provides an additional polymeric matrix for the Ag/ZnO nanophases. The abundant hydroxyl (–OH) and carboxylate-containing groups of CMC can interact with the inorganic phases through hydrogen bonding, electrostatic interactions, and coordination effects, thereby promoting their incorporation within the polymer network. Such interactions may help reduce extensive aggregation and provide a favorable aqueous environment for contact between the nanocomposite and target molecules. Similar interactions between CMC and metal-containing nanophases have been reported for other CMC-based nanocomposite systems.^28,29^

The CMC matrix may also facilitate the accessibility of aqueous Crystal violet molecules to exposed catalytic sites by providing a hydrophilic polymeric environment around the inorganic nanophases. Under visible-light irradiation, the close interfacial contact between Ag and ZnO may promote interfacial electron transfer and reduce electron–hole recombination, thereby contributing to the photocatalytic performance of the composite. This interpretation is consistent with the proposed involvement of reactive species in CV degradation, particularly ˙OH and O_2_˙^−^, as demonstrated by the selective radical-scavenging experiments. The incorporation of Ag can additionally provide electron-trapping sites, while the ZnO phase serves as a semiconductor photocatalytic component.

Furthermore, phytochemical residues originating from cinnamon may provide additional redox-active and surface-functional groups that could contribute to the antioxidant and antimicrobial characteristics of the material.^30^ Thus, the overall formation of the Ag/ZnO/CMC NC can be regarded as a multistep process involving (i) phytochemical-mediated transformation and coordination of Ag^+^ and Zn^2+^ precursor ions, (ii) nucleation and growth of Ag/ZnO nanophases, and (iii) incorporation and stabilization of the inorganic phases within the CMC matrix. The combined interactions between the cinnamon phytochemicals, Ag/ZnO phases, and CMC provide a plausible basis for the physicochemical characteristics and multifunctional performance of the resulting nanocomposite.

The proposed mechanism is supported by the combined structural, morphological, and compositional characterization results presented in the manuscript and SI; however, the individual molecular-level steps should be regarded as proposed interpretations rather than directly observed processes. Although the selected Ag/ZnO NC-to-CMC ratio exhibited high photocatalytic and antibacterial performance, the present study did not systematically evaluate the effect of nanoparticle loading on these properties. Therefore, the selected ratio should be considered a defined formulation rather than an experimentally optimized composition, and future studies should investigate a broader range of Ag/ZnO-to-CMC ratios to establish the optimum composition.

### 3.2. Characterization of nanocomposites

#### 3.2.1. UV-visible spectroscopy

**Fig. 2:**
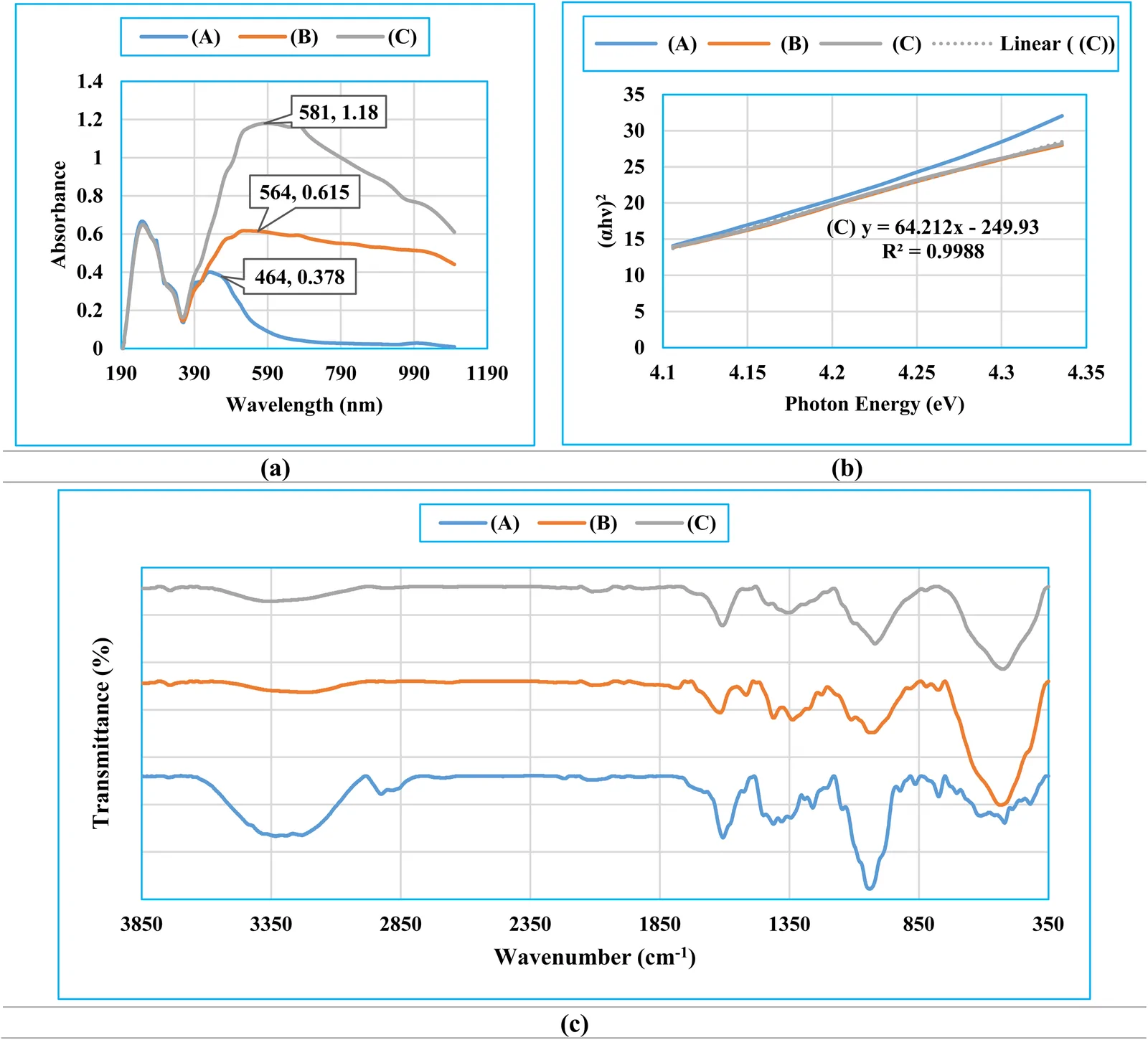
(a) UV-Vis absorption spectra of (A) cinnamon extract, (B) Ag/ZnO NC, and (C) Ag/ZnO/CMC NC, showing progressive red shifts and enhanced absorbance upon nanocomposite formation and polymer incorporation. (b) Corresponding Tauc plots used to estimate the optical band gap energies of (A) cinnamon extract, (B) Ag/ZnO NC, and (C) Ag/ZnO/CMC NC, indicating significant modulation of electronic structure due to metal–semiconductor coupling and CMC stabilization. (c) FTIR spectra of (A) cinnamon extract, (B) Ag/ZnO nanocomposite, and (C) Ag/ZnO/CMC nanocomposite, where the role of phytochemical functional groups in the reduction and stabilization of nanoparticles, the appearance of metal–oxygen bands indicating the formation of Ag–Zn phases, and the encapsulation of the nanocomposites by CMC are evident.

The UV-Vis absorption spectra of the cinnamon extract, Ag/ZnO NC, and Ag/ZnO/CMC NC are shown in Fig. 2a. The absorption spectra of the cinnamon extract contained one distinct absorption peak at 464 nm with an absorbance value of 0.378. The aforementioned absorption is related to π–π* transitions of phenolic, flavonoid, and other conjugated phytochemical compounds in the extract (Fig. 2a and Table S1). These phytochemicals may take part in the reduction, nucleation, and stabilization of metal and metal oxide nanoparticles *via* the green synthesis process. The Ag/ZnO NC sample demonstrated a noticeable bathochromic shift of the dominant absorption peak to 564 nm with an increased absorbance of 0.615. The above-mentioned changes in the absorption spectra can be explained by the formation of Ag/ZnO nanostructured clusters and the contribution of Ag-induced optical transitions, including plasmonic transitions, as was observed for Ag/ZnO-based nanocomposites synthesized using the green approach.^31^

After the addition of carboxymethyl cellulose (CMC), the Ag/ZnO/CMC nanocomposite exhibited another red shift in its absorption peak to 581 nm, with a value of absorbance 1.18. The increased and widened absorption spectrum could be attributed to the interactions at the interface between the nanophases of Ag/ZnO and the matrix of CMC, as well as due to better dispersion and functionalization of the inorganic nanophases. The same trend in the absorption behavior has been observed in other Ag/ZnO nanocomposites, based on cellulose and chitosan.^32^

The optical band-gap behavior was further investigated using the Tauc relation, ((*αhν*)^2^ = *A*(*hν* − *E*_g_)), where (*α*) is the absorption coefficient, (*hν*) is the photon energy, (*A*) is a proportionality constant, and (*E*_g_) is the apparent optical band-gap energy. The optical band-gap energy of the Ag/ZnO/CMC NC was estimated using the Tauc relation, (*αhν*)^2^ = *A*(*hν* − *E*_g_), and the corresponding plot is presented in Fig. 2b. The linear portion of the absorption edge yielded the regression equation *y* = 64.212*x* − 249.93, with an excellent correlation coefficient (*R*^2^ = 0.9988). Extrapolation of the linear region to (*αhν*)^2^ = 0 gave an apparent optical band-gap energy of approximately 3.89 eV. Thus, the UV-Vis data obtained and the subsequent Tauc analysis prove that the combination of Ag/ZnO within the CMC medium creates a distinct absorption spectrum, confirming the successful synthesis of the desired Ag/ZnO/CMC phytogenic nanocomposite.

#### 3.2.2. FTIR spectroscopy

The FTIR spectroscopy analysis showed the progressive formation of the Ag/ZnO and Ag/ZnO/CMC nanocomposites using cinnamon extract (Fig. 2c & Table S2). The extract showed a broad O–H stretching band at 3375–3227 cm^−1^, aromatic C=C stretching at 1606–1522 cm^−1^, and intense C–O/C–O–C stretching at 1258–1019 cm^−1^, indicative of the presence of polyphenols, flavonoids, and polysaccharides. In the formation of the nanocomposite, changes were observed in the Ag/ZnO spectrum, showing attenuation and shifts in these bands, especially the O–H band at 3216 cm^−1^ and the aromatic bands at 1609–1511 cm^−1^. The appearance of new low-frequency bands in the 647–419 cm^−1^ region is consistent with metal–oxygen vibrations, particularly Zn–O stretching associated with the ZnO phase, supporting the formation of the inorganic component of the nanocomposite. This encapsulation within the CMC matrix was accompanied by further spectral stabilization, as indicated by the intensified O–H stretching vibration at 3357 cm^−1^ and the characteristic carboxylate-related bands at approximately 1614–1602 and 1434 cm^−1^, respectively, indicating that the carboxylate groups of CMC are involved in interactions with the inorganic nanophases. The persistence of the polysaccharide backbone absorption at 1251–1021 cm^−1^ and the metal–oxygen absorption at 665–395 cm^−1^ indicates the structural integrity of the system. These results are in close agreement with previous studies on CMC-stabilized Ag and Ag/ZnO nanocomposites, in which the presence of the polymer improves dispersion, stability, and functional properties of the systems.^33^ Compared to the uncoated systems, the Ag/ZnO/CMC nanocomposite exhibits better structural arrangement and interfacial interactions, confirming its potential in high-performance antimicrobial and environmental applications.

#### 3.2.3. XRD analysis

**Fig. 3:**
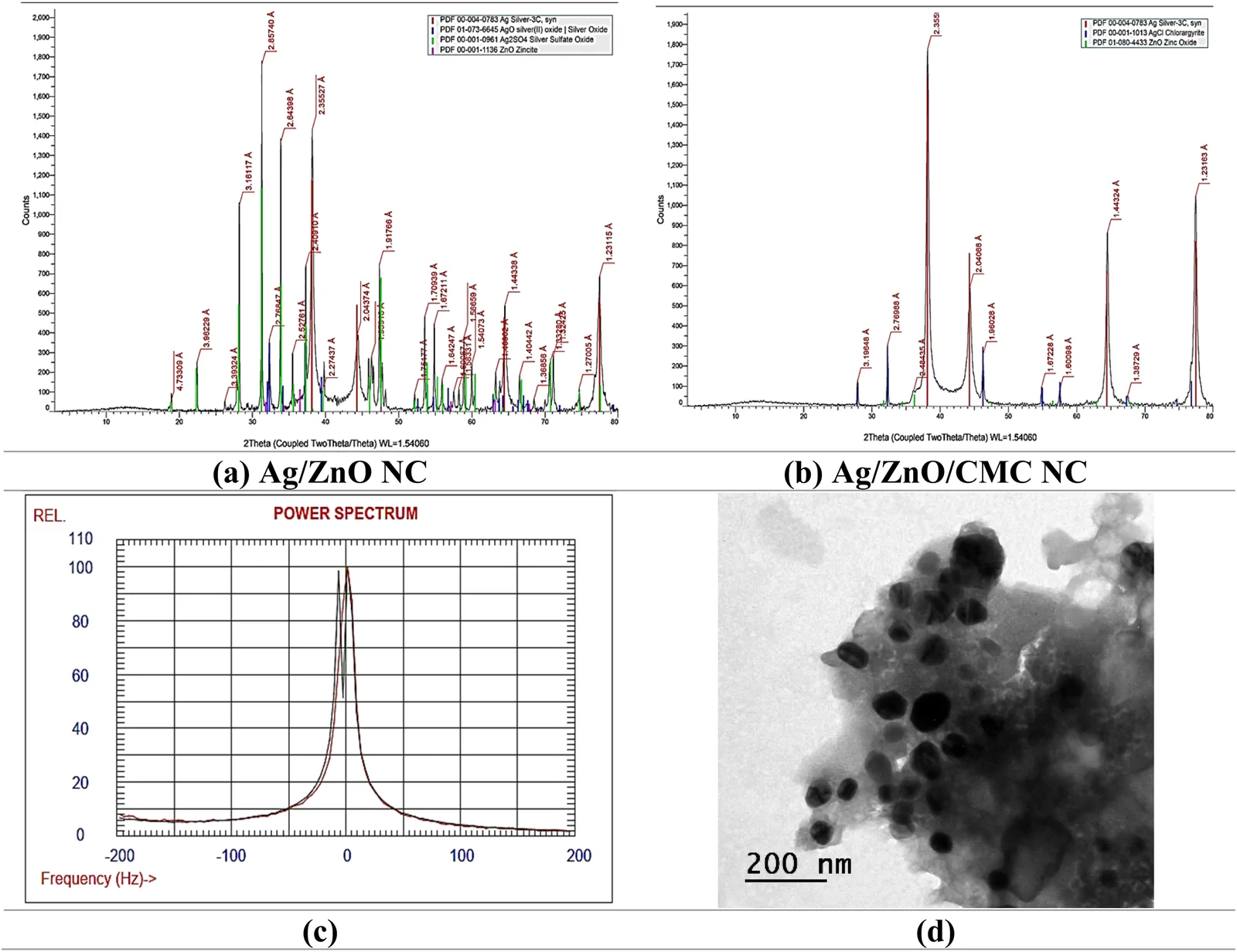
X-ray diffraction (XRD) patterns of (a) Ag/ZnO NC and (b) Ag/ZnO/CMC NC recorded in the 2*θ* range of 10–80° using Cu Kα radiation (*λ* = 1.5406 Å), showing the multiphase crystalline nature of the materials with dominant metallic Ag reflections and the presence of Ag_2_SO_4_, ZnO (zincite), and AgO in Ag/ZnO, as well as Ag, AgCl, and wurtzite ZnO phases embedded within the amorphous CMC matrix in Ag–ZnO/CMC. (c) Zeta potential of Ag/ZnO/CMC NC, indicating surface stabilization and dispersion behavior mediated by the carboxymethyl cellulose (CMC) matrix and phytochemical capping agents. (d) TEM micrograph of the prepared Ag/ZnO/CMC NC, illustrating quasi-spherical, ellipsoidal, and polygonal-shaped Ag/ZnO nanostructures with dimensions of 20–80 nm dispersed into the amorphous CMC medium. The TEM picture reflects the successful dispersion of the nanostructures, effective stabilization using polymer, and the close interaction between Ag and ZnO phases.

The XRD patterns of the Ag–ZnO nanocomposite also revealed the presence of a highly crystalline multiphase material (Fig. 3a). The XRD pattern indicates the presence of metallic Ag^0^ and crystalline ZnO (zincite) as the principal inorganic phases. The characteristic Ag^0^ reflections, particularly the intense peak at 2*θ* ≈ 38.18° corresponding to the (111) plane of face-centered cubic Ag, support the formation of metallic silver, while the additional reflections are consistent with the ZnO phase. The highest intensity peak was recorded at 2*θ* = 31.279° with *d* = 2.857 Å. This can be considered as the standard peak. The highest intensity peak at 38.180° (*d* = 2.355 Å) is related to the (111) planes in the face-centered cubic silver phase, as is the case in the previously prepared Ag–ZnO nanocomposites.

In the Ag–ZnO/CMC nanocomposite, XRD analysis further supports the existence of inorganic crystals that have been well incorporated into the polymeric medium (Fig. 3b). On a semi-quantitative basis, it has been identified that the major constituents of the crystals, along with metallic silver, are silver chloride (AgCl) and wurtzite ZnO, in a proportion of 45.9%, 35.7%, and 18.4%, respectively. The major peaks of metallic silver lie on the plane (111) at 2*θ* of 38.226°, whereas other prominent peaks lie at 32.222° and 27.889° for AgCl, which belong to planes (200) and (111), respectively. AgCl is generally formed frequently in polysaccharide-mediated syntheses because of the interaction between Ag^+^ ions and chloride ions and generally increases the stability of Ag-containing nanocomposites.^33,34^ The absence of any sharp diffraction peak attributable to carboxymethyl cellulose confirms its predominantly amorphous nature, which is consistent with its stabilizing role in resisting crystal growth and yielding a peak broadening (FWHM = 0.200–0.400°). Overall, the coexistence of Ag, AgCl, and ZnO phases within the CMC matrix is consistent with literature reports and is expected to favor synergistic effects to deliver enhanced antimicrobial and photocatalytic applications.^34,35^

Semi-quantitative analysis identifies the major crystalline components as metallic Ag (45.9%), silver chloride (AgCl, 35.7%), and wurtzite ZnO (18.4%). For metallic silver, its most intense peak was situated at 2*θ* = 38.226° and ascribed to reflection from the (111) plane. Other strong reflections at 32.222° and 27.889° were assigned to the (200) and (111) planes of AgCl, respectively. The formation of AgCl is widely encountered in polysaccharide-mediated syntheses due to the interaction of Ag^+^ with residual chloride ions, and it usually enhances the functional stability of Ag-based nanocomposites.^33,34^ The absence of distinct sharp diffraction peaks attributable to CMC is consistent with the predominantly amorphous nature of the polymer matrix. The broadening of the inorganic reflections may reflect the nanoscale nature of the crystalline domains and microstructural effects associated with their incorporation into the polymer matrix. Generally, the coexistence of Ag, AgCl, and ZnO phases within the CMC matrix is consistent with literature reports and is expected to favor synergistic effects to deliver enhanced antimicrobial and photocatalytic applications.^33,34^

#### 3.2.4. Zeta potential analysis

The surface charge and colloid stability of the fabricated Ag/ZnO/CMC NC were characterized through zeta potential, and the results are shown in Fig. 3c. The average electrophoretic mobility and zeta potential values observed for the fabricated NC were found to be −0.12 MU and −3.69 mV, respectively. The surface negativity indicates effective stabilization of the nanocomposites by CMC and plant-derived chemical moieties present in cinnamon extract, including negatively charged groups such as hydroxyl (–OH) and carboxylate (–COO^−^). The negative value of zeta potential suggests the existence of electrostatic repulsive forces between the dispersed particles, which enhance the stability of the dispersion medium and prevent nanoparticle aggregation. While the value of zeta potential obtained was not very high as compared to other well-stabilized colloidal suspensions (normally above −30 mV), the Ag/ZnO/CMC NC possessed adequate colloidal stability because of the joint influence of electrostatic repulsion and steric stabilizing effects provided by the CMC polymer. The sterical resistance offered by the polymeric nature of CMC prevented particle aggregation and improved nanoparticle dispersion in aqueous media. Moreover, the addition of CMC to nanoparticles of Ag/ZnO could have caused the formation of a hydrated polymer layer, which facilitated suspension uniformity and prevented nanoparticle-to-nanoparticle contact. This observation is consistent with the findings made by Lungu *et al.*,^33^ where it was shown that nanocomposites of Ag/ZnO capped with CMC had better colloidal stability and higher antimicrobial activity. The slightly negative value of the zeta potential can also be significant in the photocatalytic and antibacterial properties of the material. The negatively charged surface of nanocomposites allows for electrostatic attraction to positively charged dyes like Crystal violet, contributing to the increased efficiency of photocatalytic decomposition of the dye. In addition, better dispersion stability ensures that there is more active surface area on the surface of the nanocomposite and, therefore, better ROS production upon illumination with visible light. In summary, the analysis of zeta potential confirms the success of the surface modification and stabilization of the Ag/ZnO nanoparticles in the CMC matrix.

#### 3.2.5. Transmission electron microscopy (TEM)

TEM analysis of the Ag/ZnO/CMC NC (Fig. 3d) showed that the NCs comprised nanoparticles with mainly quasi-spherical, elliptical, and polygonal shapes. The size distribution of the nanoparticles was analyzed quantitatively through TEM micrograph, where 30 nanoparticles were measured. Analysis of 30 individual nanoparticles yielded particle diameters of 32.46–128.42 nm, with most particles distributed between approximately 40 and 75 nm and a median diameter of approximately 52.7 nm (Fig. S1). Variations in electron contrast were observed within the nanocomposite, indicating differences in electron density that are consistent with the presence of inorganic nanophases; their elemental identity was supported by the corresponding EDX analysis. The nanoparticles were dispersed in an amorphous gray matrix, which was believed to be due to the CMC component. Particle agglomeration was noted; however, there were still many single particles discernible in the CMC matrix. The close spatial association of the inorganic nanophases suggests the formation of Ag/ZnO interfaces, which may facilitate interfacial interactions relevant to the photocatalytic behavior of the composite.

#### 3.2.6. Scanning electron microscopy (SEM)

**Fig. 4:**
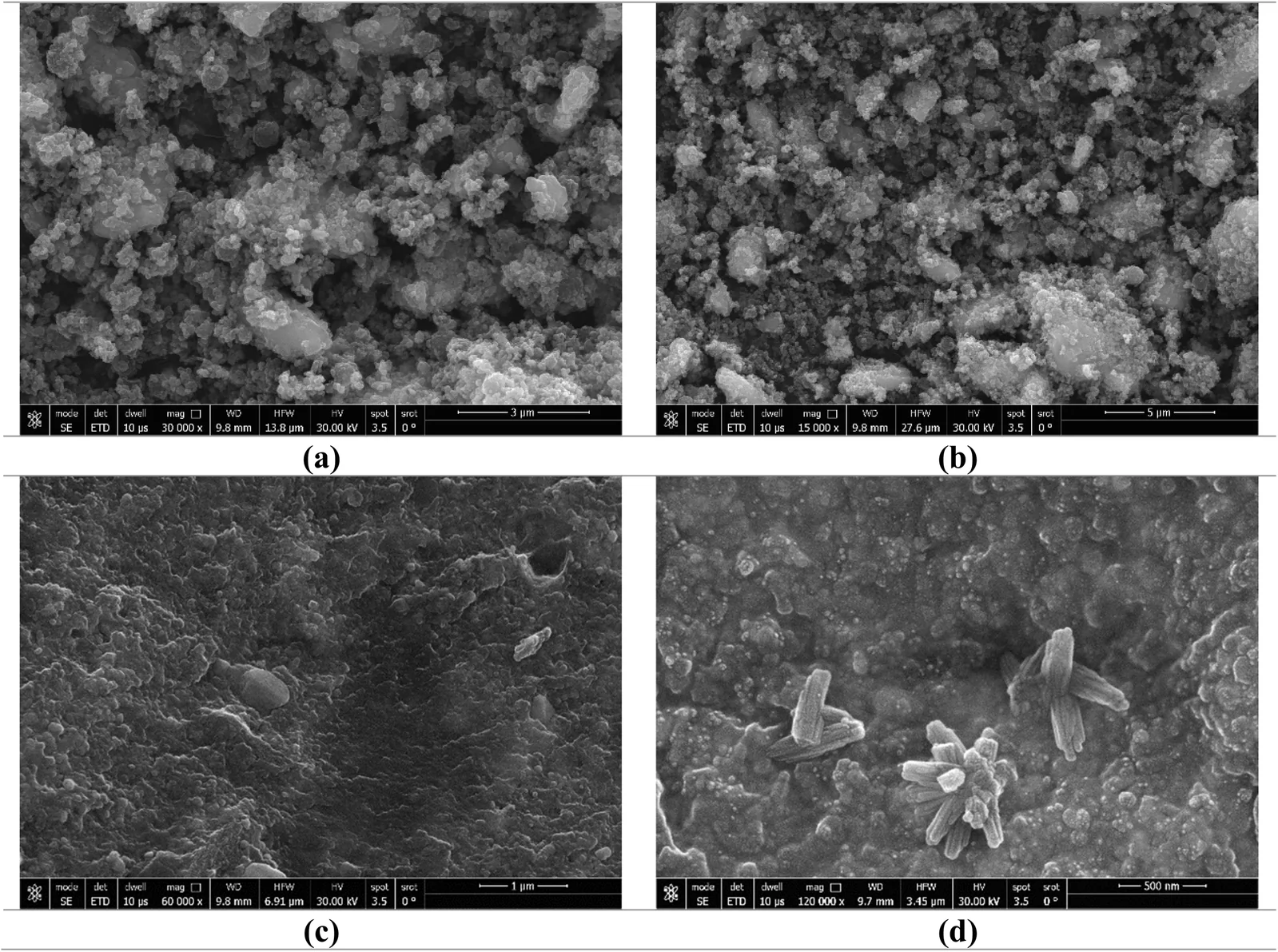
SEM micrographs of Ag/ZnO (a and b) and Ag/ZnO/CMC nanocomposites (c and d).

The surface morphology of the Ag/ZnO nanocomposite was examined by SEM analysis to interpret the influence of carboxymethyl cellulose (CMC) on Ag and Zn nanoparticles' growth and assembly. The SEM images of the pristine Ag/ZnO NC (Fig. 4a and b) indicate the presence of rough and densely clustered particles of irregular shape and large size. Moreover, the particles appear to be poorly dispersed and have a large extent of agglomeration, indicated by the Ag/ZnO nanoparticles' tendency to densely aggregate at a magnification scale of 3 µm and 5 µm. The tendency of Ag and Zn nanoparticles to agglomerate and aggregate upon nucleation can be related to their relatively high surface energy; therefore, the particles tend to interact and favor uncontrolled growth in the lack of a sufficient surface capping and modifying agent. The surface morphology of Ag and Zn nanoparticles is rough and poorly dispersed; this has a significant effect on their surface functionality.

Conversely, the presence of CMC causes a significant morphological change in the Ag/ZnO/CMC nanocomposite (Fig. 4c and d). Observations from the SEM images indicate the presence of distinct flower-like and rod-shaped crystalline morphology uniformly distributed in a continuous polymeric matrix. On the 500 nm level, the morphology shows a bundle of highly elongated nanorods oriented around a central nucleus, which implies a controlled anisotropic growth process. Additionally, the hydroxyl and carboxylate groups found in the CMC contribution played an essential role in the coordination of the metal ions. In particular, the high density and uniformity reflected in the Ag/ZnO/CMC matrix confirms the successful encapsulation of the inorganic phases by the presence of the matrix.

The structural change through the CMC-mediated pathway is extremely helpful from a functional viewpoint. The flower-like structure with a hierarchical morphology offers a much larger surface area with a higher density of accessible active sites in comparison to the Ag/ZnO counterpart in the aggregated form. Moreover, the strong interfacial interaction between the Ag/ZnO nanoparticles and the CMC matrix improves the degree of dispersion and prevents the occurrence of any secondary agglomeration. The collective advantages are expected to benefit the efficiency of electron transfer, ion-release behavior, and interfacial activity, which are collectively important for acquiring superior antimicrobial, photocatalytic, and environmental applications. The present finding is in agreement with the previously documented polysaccharide-mediated metal nanocomposites, which also demonstrate the importance of the CMC unit in determining the nanostructure architecture.

#### 3.2.7. EDX and elemental mapping analysis

**Fig. 5:**
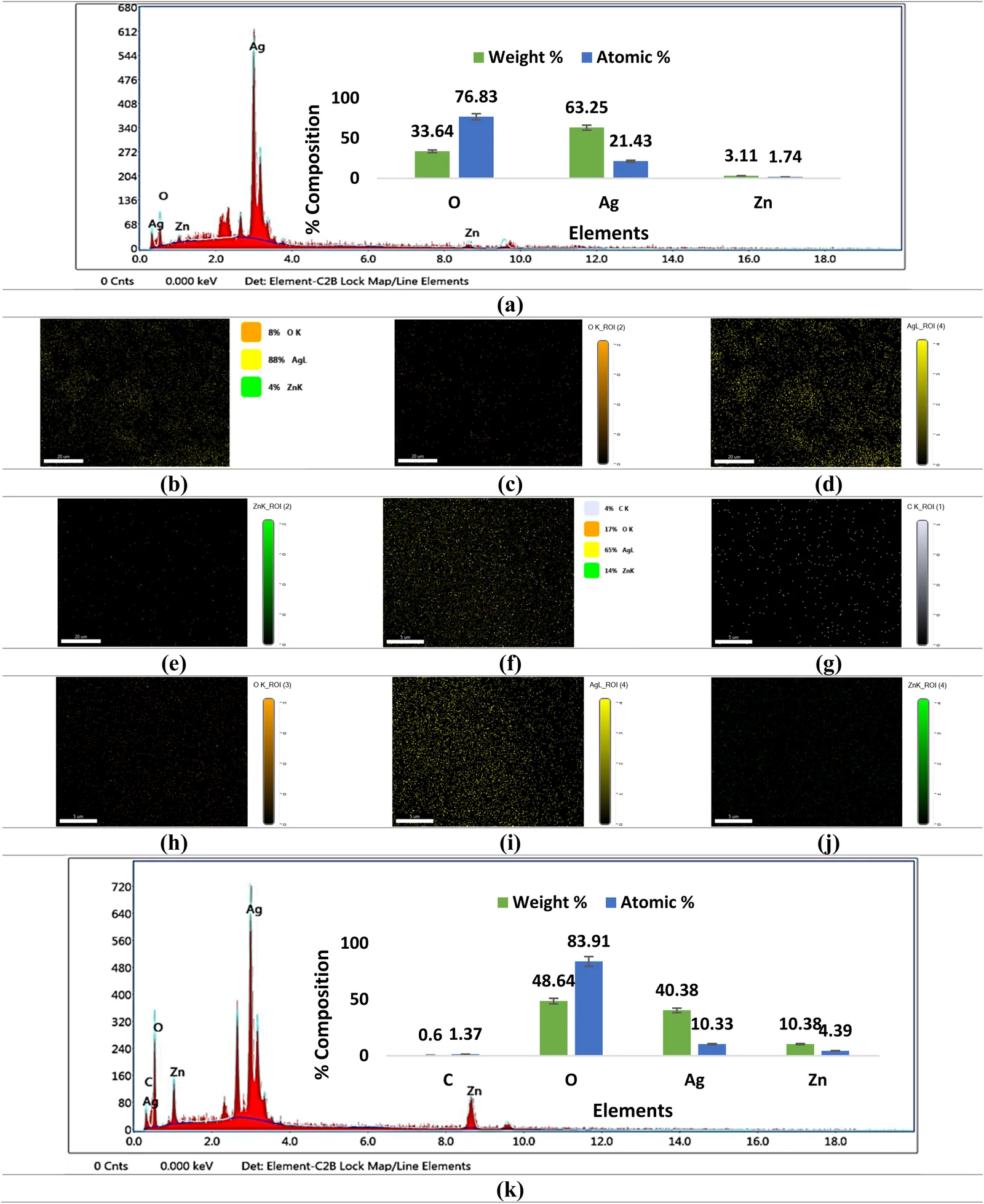
EDX spectra and elemental mapping analysis of Ag/ZnO and Ag/ZnO/CMC nanocomposites. (a) EDX spectrum of Ag/ZnO NCs; (b–e) elemental mapping of Ag/ZnO NCs showing the distribution of (b) combined elements, (c) O, (d) Ag, and (e) Zn; (f–j) elemental mapping of Ag/ZnO/CMC NCs showing the distribution of (f) combined elements, (g) C, (h) O, (i) Ag, and (j) Zn; (k) EDX spectrum of Ag/ZnO/CMC NCs. CMC refers to carboxymethyl cellulose.

Energy-dispersive X-ray spectroscopy (EDX) and elemental mapping were used to determine the elemental composition and spatial distribution of the principal elements in the Ag/ZnO and Ag/ZnO/CMC nanocomposites. The EDX spectra of the respective nanocomposites are represented in Fig. 5, while the results of the quantitative analyses are included in Tables S3 and S4.

The EDX spectrum of the Ag/ZnO nanocomposite (Fig. 5a) shows the presence of elements such as silver (Ag), zinc (Zn), and oxygen (O). The absence of spectral lines of impurities in the EDX spectrum is worth noting. As evident from the table (Table S3), Ag is prominent in the composite, which makes up 63.25 wt% (21.43 at%), thus verifying the formation of Ag-rich nanostructures. The appearance of the signal corresponding to Zn (3.11 wt%) in the composite confirms the effective integration of Zn into or onto the Ag matrix. The oxygen signal (33.64 wt%) is consistent with the presence of the ZnO phase and oxygen-containing surface species. However, EDX alone cannot distinguish lattice oxygen from surface hydroxyl or other oxygen-containing species.

For the Ag/ZnO/CMC nanocomposite, the EDX analysis shown in Fig. 5k identifies the co-existent peaks for carbon (C), which verifies the successful surface modification using carboxymethyl cellulose. Based on the EDX analysis in Table S3, the weight% of Ag reduces to 40.38% with the addition of the polymeric matrix, while that of Zn rises to 10.38%, indicating the increased relative contribution of Zn in the analyzed CMC-containing region. The dominant weight% increase in oxygen (48.64%) is attributable to the large amount of hydroxyl and carboxyl groups in the carboxymethyl cellulose, which serve as the coordinating and capping agents for the metal ions. The presence of the carbon peak (0.60%) also verifies the successful modification using the polysaccharide matrix. In conclusion, the results obtained using the EDX analysis affirm the presence of both nanocomposites, as well as the effectiveness of CMC in surface chemistry modification in the Ag/ZnO system.

An analysis of elemental mapping was carried out in an attempt to understand the spatial combination of the elements in the resulting nanocomposites. The elemental maps in Fig. 5b–e show that Ag, Zn, and O signals are distributed throughout the analyzed area without obvious large-scale elemental segregation. The data in Table S4 presents an accurate depiction of the composition, composed of 88% Ag, followed by 8% O and 4% Zn, which aligns with the EDX analysis.

In the case of the Ag/ZnO/CMC nanocomposite, Fig. 5f–j shows the elemental mapping, which highlights a well-dispersed distribution of Ag and Zn embedded within an oxygen- and carbon-rich matrix. The relatively homogeneous carbon signal in Fig. 5g is consistent with the presence of CMC throughout the analyzed region; however, elemental mapping alone does not establish a continuous or uniform polymer coating around individual nanoparticles. Table S4 shows that Ag remains the predominant element at 65%, while Zn content increased to 14%, suggesting that CMC facilitates strong interaction and stabilizes Zn species. The higher oxygen content of 17% further reflects the contribution of functional groups of CMC and metal–oxygen bonding. The absence of pronounced large-scale elemental clustering in the analyzed areas indicates a relatively homogeneous spatial distribution of the detected elements, supporting the successful incorporation of the inorganic components within the CMC-containing matrix. More specifically, the key significance of the enhanced elemental homogeneity of the Ag/ZnO/CMC nanocomposite lies in the pivotal function of the CMC dispersing agent in terms of furthering stability.

#### 3.2.8. X-ray photoelectron spectroscopy (XPS) analysis

**Fig. 6:**
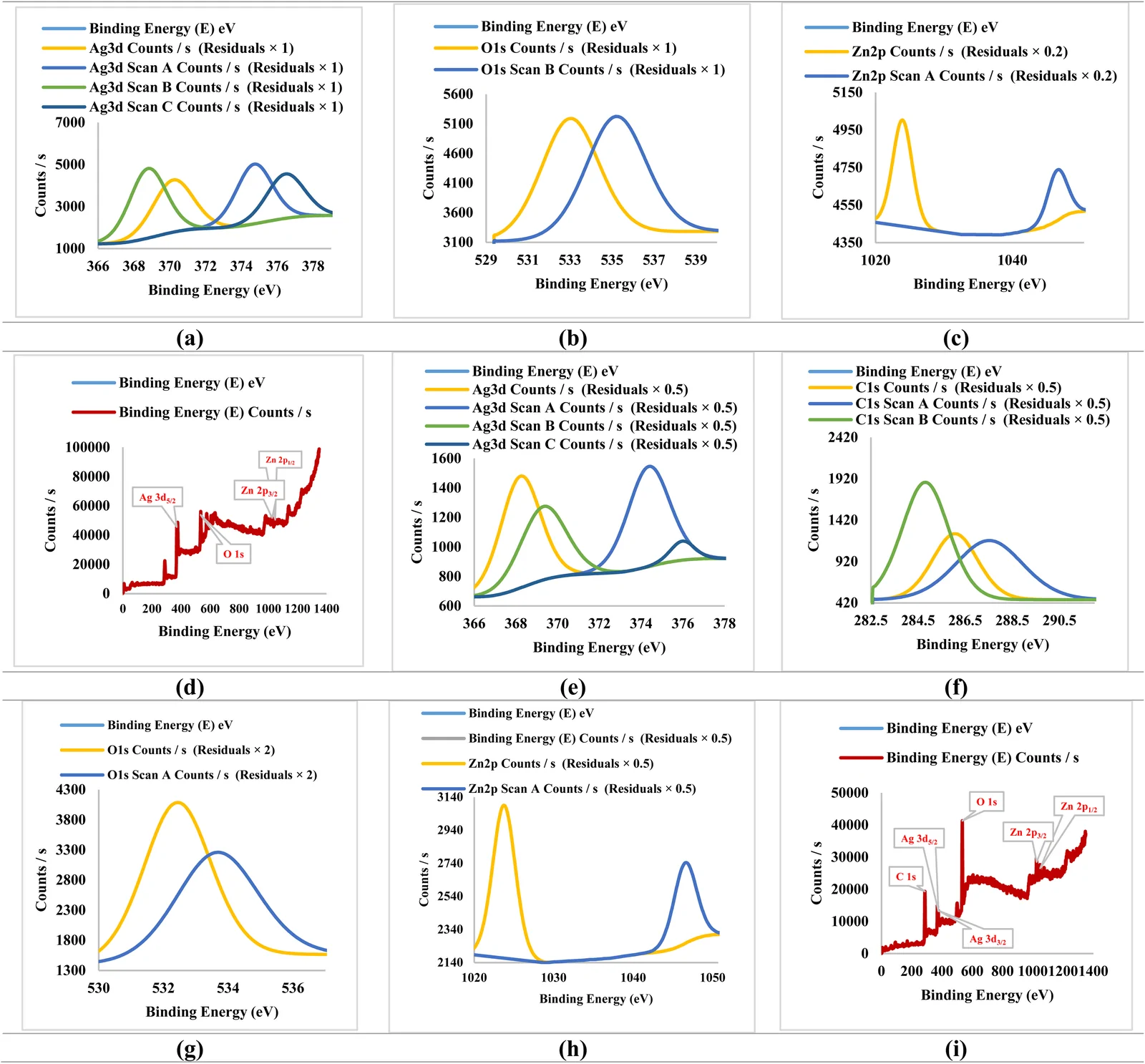
X-ray photoelectron spectroscopy (XPS) analysis of (a–d) Ag/ZnO NC and (e–i) Ag/ZnO/CMC NC: high-resolution spectra of Ag 3d (a and e), O 1s (b and g), Zn 2p (c and h), C 1s (f), and corresponding XPS survey spectra (d and i), showing the surface elemental composition and chemical states of the Ag, Zn, O, and C components.

The surface chemical composition and oxidation state of the prepared Ag–ZnO nanocomposite were analyzed using XPS, and the corresponding high-resolution spectra are shown in Fig. 6a–d. The survey scan spectrum shows clear peaks corresponding to Ag, Zn, and O, along with a trace amount of adventitious carbon, ensuring that the prepared Ag–ZnO does not contain any impurities. The Ag 3d spectrum exhibits the characteristic Ag 3d_5/2_ and Ag 3d_3/2_ components at approximately 368–369 and 374–376 eV, respectively, consistent predominantly with metallic Ag^0^. No additional Ag oxidation state is assigned because the present XPS data do not provide sufficient evidence for a distinct oxidized Ag species.

These values are quite comparable with other reported Ag–ZnO nanocomposites where Ag^0^ is the major surface component playing a significant role in the charge transfer process at the interface.^32,36^ The Zn 2p spectrum exhibits two prominent components at approximately 1023.9 eV (Zn 2p_3/2_) and 1046.6 eV (Zn 2p_1/2_), consistent with Zn^2+^ in the ZnO lattice and supporting the presence of the ZnO phase. The O 1s core-level spectrum has two distinguishable peaks at approximate values of 532.7 eV and 534.8 eV for lattice oxygen (Zn–O) and surface-adsorbed oxygen species or hydroxyl group components, respectively. The higher-binding-energy O 1s component can be attributed to surface hydroxyl groups and/or adsorbed oxygen-containing species; however, oxygen vacancies cannot be conclusively assigned from the O 1s spectrum alone.

For the Ag–ZnO/CMC nanocomposite, Fig. 6i (XPS survey spectrum) shows an evident increase in carbon content with concomitant signals of Ag, Zn, and O, suggesting that the incorporation of a carboxymethyl cellulose matrix was successful. In the case of Ag 3d core-level spectrum, Fig. 6e, the binding energies are positioned at ∼367.9–369.0 eV and ∼374.4–376.0 eV, which are slightly shifted to the lower energy sides compared to the Ag–ZnO sample. This shift indicates electronic interaction between silver nanoparticles and functional groups of CMC, especially carboxylate and hydroxyl moieties, partial electron donation to Ag, and enhancement of interfacial stabilization.^32,36^

The spectra of the C 1s core levels (see Fig. 6f) can be curve–fit with the peak at ∼284.7 eV for the C–C/C–H components and with the peak at 285.5–287.1 eV for the remaining components to emphasize the polysaccharide chain for the CMC and the metal components. The peak for the Zn 2p core levels appeared at ∼1023.8 and 1046.6 eV. These Zn 2p binding energies remain consistent with Zn^2+^ in the ZnO lattice, indicating that incorporation of CMC does not result in an apparent change in the Zn oxidation state. Simultaneously, the O 1s core-level spectra indicates the presence of metal–oxygen bonds as well as oxygen functional groups in CMC, thus supporting a relatively high level of interfacial interactions among ZnO, Ag, and the biopolymer matrix under test. These results are in accordance with those found for cellulose-based Ag–ZnO nanocomposites, wherein polymer functional groups are known to increase dispersions, stabilize active surface sites, and increase surface reactivities.^32,36,37^ These findings, in turn, provide credible evidence in support of the successful formation of interactive Ag–ZnO and Ag–ZnO/CMC nanocomposites with a major contribution of CMC towards controlling surface functionalities and possibly enhancing photonic and biological properties.

#### 3.2.9. Textural and surface desorption properties

##### 3.2.9.1. BET surface area and porosity analysis

**Fig. 7:**
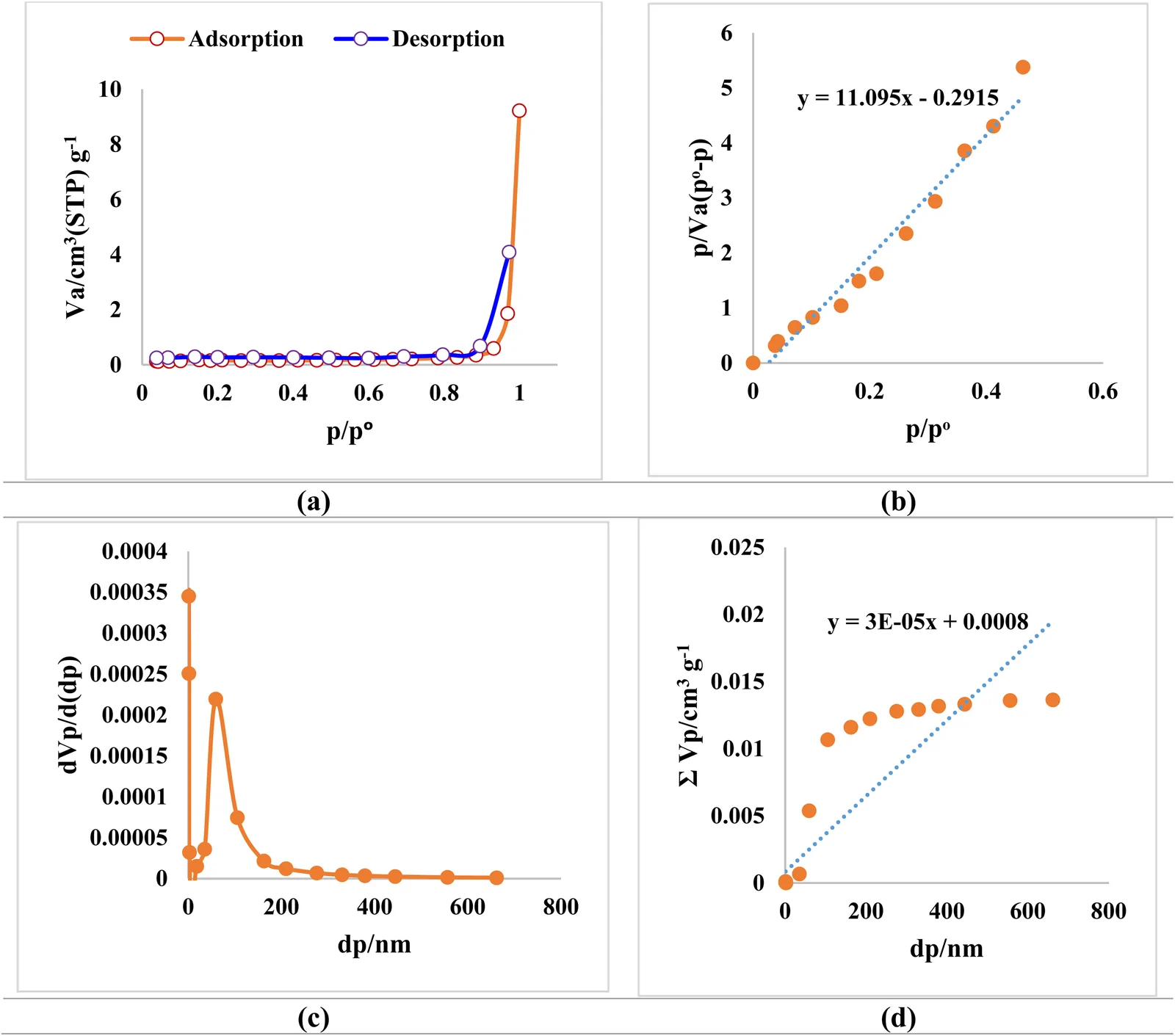
BET and BJH surface porosity analysis of the synthesized Ag/ZnO/CMC nanocomposite (NC). (a) Nitrogen adsorption–desorption isotherm illustrating the adsorption behavior and porous nature of the Ag/ZnO/CMC NC. (b) BET linear fitting plot showing the correlation between relative pressure (*p*/*p*_0_) and *p*/[*V*_a_(*p*_0_ − *p*)]; the slope and intercept of the fitted equation were used to determine the specific surface area, BET constant, and monolayer adsorption capacity. (c) BJH pore size distribution curve depicting the variation of differential pore volume d*V*_p_/d(*d*_p_) with pore diameter (*d*_p_), providing insight into the mesoporous structure of the nanocomposite. (d) Cumulative pore volume (Σ*V*_p_) as a function of pore diameter, demonstrating the pore volume distribution and overall porosity characteristics of the Ag/ZnO/CMC NC.

The textural features and the porous nature of the obtained Ag/ZnO/CMC NC were determined based on BET and BJH analyses, as shown in Fig. 7. The isotherm profile of nitrogen adsorption–desorption (Fig. 7a) showed a typical type IV isotherm with an evident hysteresis loop at high relative pressures (*P*/*P*_0_ > 0.7), which reflects the existence of mesoporosity within the NC material. The progressive growth in nitrogen adsorption under low relative pressures and then a rapid rise at near-saturation pressure implies multilayer adsorption with capillary condensation in interconnected pores. This adsorption feature is common for porous nanohybrid materials with heterogeneous surfaces and internal cavities. In this regard, type IV mesoporous isotherms were found in the case of Ag@ZnO/cellulose nanocomposites fabricated for dye removal and antibacterial purposes, where increased porosity had a considerable contribution to enhanced adsorption performance and surface activity.^38^

According to the BET linear fitting graph (Fig. 7b), there was a good linearity in relative pressure *versus P*/[*V*_a_(*P*_0_–*P*)]. Therefore, this indicates the validity of the use of the BET equation for the determination of the surface area. Based on the continuity of the adsorption curve over a broad range of pressures, it can be concluded that adsorption sites are abundant, as well as the availability of pores, due to the addition of Ag and Zn nanoparticles to the CMC system. The possible cause of the formation of pores is the dispersion of the nanoparticles, the rearrangement of the polymer chains, and interaction at the interface of metallic nanoparticles with hydroxyl/carboxyl groups of carboxymethyl cellulose (CMC). Similar findings have been reported by Viswanathan *et al.*^39^

The BJH pore size distribution graph (Fig. 7c) confirmed that the Ag/ZnO/CMC NC displayed primarily mesoporous characteristics, with most of the pores having diameters lying between 5 and 20 nm. The emergence of numerous pores with dimensions ranging between 5.0 and 6.8 nm indicates mesoporosity, while the existence of a broader range of pores that extend to higher diameters indicates macroporosity as well. The benefit of having this arrangement of pores is its efficiency in providing molecular transport to reach surface sites for adsorption and catalysis processes.

The cumulative pore volume plot (Fig. 7d) served as an additional indication regarding the porosity of the acquired nanocomposite, as it showed that the cumulative pore volume was around 1.36 × 10^−2^ cm^3^ g^−1^, while the cumulative surface area was close to 0.45 m^2^ g^−1^. While the obtained value of the surface area can be considered relatively moderate compared to porous inorganic materials, the achieved porosity level is still adequate for effective surface interactions and adsorption reactions. Such relatively low values of the surface area might result from the formation of pores being partially blocked by agglomerated nanoparticles and the dense packing of the polymer CMC matrix.

Furthermore, the gradual decrease in pore size from macropores (>50 nm) to mesopores and micropores (<10 nm) implies the creation of an uneven structure of the porous material. Heterogeneity of the pore size distribution is advantageous for applications due to the ability of larger pores to promote mass transfer, while smaller pores can have a larger number of active surface sites. Formation of such pores may be associated with solvent evaporation, nucleation of nanoparticles, and immobilization of the Ag/ZnO nanoparticles in the CMC biopolymer matrix. Hierarchical porosity was shown before for bimetallic biochar nanocomposites with enhanced photocatalytic decomposition and antimicrobial properties.^39^ Generally, the BET and BJH results indicated that the prepared Ag/ZnO/CMC nanocomposite material is hierarchically structured with easily accessible pores, medium pore volume, and interconnected porous domains. This type of structure has proven to be very suitable for adsorption, photocatalytic, antimicrobial, and environmental purification purposes since it facilitates better diffusion and enhances surface interaction.

##### 3.2.9.2. Temperature-programmed desorption (TPD)

**Fig. 8:**
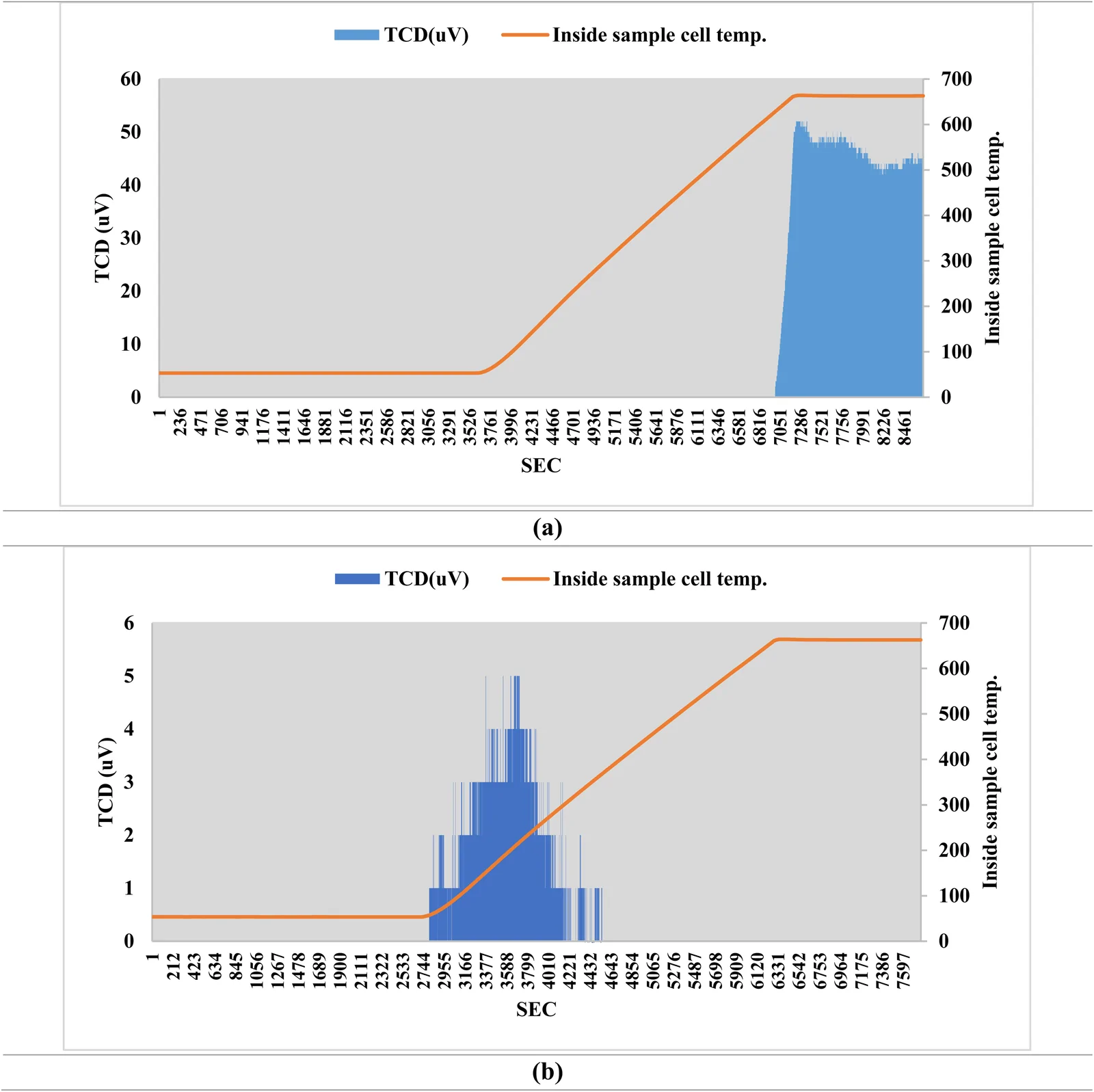
Temperature-programmed desorption (TPD) analysis of the synthesized Ag/ZnO/CMC nanocomposite (NC): (a) CO_2_-TPD profile illustrating the distribution and strength of basic surface-active sites, and (b) NH_3_-TPD profile showing the acidity and distribution of acidic surface sites of the Ag/ZnO/CMC NC.

CO_2_-TPD and NH_3_-TPD studies were performed to analyze the surface acid–base properties of the Ag/ZnO/CMC NC, as shown in Fig. 8. These studies offer significant insights into the location, amount, and intensity of the surface-active sites that play a vital role in determining the adsorption capacity and catalytic activity of the nanocomposite.

Three clear basic desorption peaks were observed from the CO_2_-TPD analysis (Fig. 8a), representing weak, medium, and strong basic sites present on the Ag/ZnO/CMC NC surface. The total basicity of the nanocomposite was found to be 310.12 µmol g^−1^, suggesting that there exist sufficient amounts of active electron donor sites that can react with the acidic probe like CO_2_. The first peak of desorption was recorded at 108.6 °C, corresponding to weak Brønsted basic sites, which contribute 10.96 µmol g^−1^ or 3.5% of the total basicity. Weak Brønsted basic sites are usually due to surface hydroxyl and oxygen-containing groups attached to CMC.

The second peak of desorption centered around 412.3 °C was indicative of the major contributor to basicity, contributing to 163.63 µmol g^−1^ (52.8%). This second peak is known to correspond to the strength of the Lewis basic sites of medium basicity that arise due to interactions between Ag/ZnO-based particles and oxygen-containing functional groups present on the CMC support structure. This prevalence of medium basicity sites indicates the availability of numerous electron-dense sites that favor adsorption and catalysis. Similar behavior has been observed in the silver-based metal oxide composite materials in which silver nanoparticles and metal oxides exhibit greater surface reactivity by means of reactive oxygen species creation.^40^

Third desorption peaks were also found at 632.1 °C, attributed to strong structural basic sites, whose desorption quantity is 135.53 µmol g^−1^ (43.7%). Strong basic sites at high temperatures are often associated with low-coordinated anions O^2−^, oxygen vacancies, and defects in the Ag/ZnO/CMC structure. Relatively high amounts of strong basic sites reveal strong CO_2_ adsorption behavior; thus, the inclusion of zinc compounds appears to be quite essential for stabilizing active oxygen-rich centers in the Ag/ZnO/CMC structure. It was also confirmed that the presence of both medium and strong basic sites (more than 96% of basicity) demonstrates highly reactive properties of the nanocomposite surface.

Further analysis of the NH_3_-TPD curve (Fig. 8b) indicated the presence of acidic surface properties of the Ag/ZnO/CMC NC. The desorption pattern consisted of three stages representing weak, moderate, and strong acidic sites with an overall acidity level of 358.54 µmol g^−1^. The initial desorption stage was characterized by the desorption of ammonia from weak Brønsted acidic sites at 134.4 °C, representing 162.77 µmol g^−1^ (45.4%) of the total acidity level. Weak acidic sites are generally attributed to surface hydroxyl groups, protonated oxygen groups, and ammonia absorbed *via* hydrogen bonds onto the polymer backbone.

Second peak of desorption appeared at 432.8 °C, which referred to medium-strength Lewis acidity sites having a desorption quantity of 129.08 µmol g^−1^ (36.0%). Such acidic sites can be considered due to coordinatively unsaturated Ag^+^ and Zn^2+^ metal ions with an electron-pair acceptor function. The significant level of Lewis acidity reflects the formation of high-energy metallic complexes within the catalyst structure, providing higher surface polarization and adsorption properties. Similar results have been obtained for silver oxide catalysts with metal oxides, where the synergic effect of metallic nanoparticles with a support matrix has created efficient acidic sites, contributing to better oxidation rates.^41^

The third desorption peak at 643.5 °C was indicative of strong acidic sites, which had a desorption capacity of 66.69 µmol g^−1^ (18.6%). Strong acid sites are generally indicative of structural defects, metal–oxygen coordination, and ammonia complexes tightly held by the nanocomposites. High temperature desorption peaks indicate that there are thermally stable acidic sites, which can hold on to adsorbates even when subjected to higher temperatures. Strong acidic sites are favorable when performing electron transfer reactions during catalysis.

Both the CO_2_-TPD and NH_3_-TPD analyses illustrate that the Ag/ZnO/CMC NC displays an amphoteric surface, which is attributed to the combination of acidic and basic active sites. This equal acid-base combination is very beneficial for a wide variety of applications, such as adsorption, photocatalysis, antibacterial behavior, and environmental protection. The simultaneous presence of weak, moderate, and strong active sites leads to enhanced surface reactivity and increases the interactions between the nanocomposite and different organic or inorganic compounds. Additionally, the high content of moderate and strong surface-active sites indicates that the inclusion of Ag and Zn nanoparticles in the CMC matrix has resulted in the formation of defective and chemically reactive surfaces.

### 3.3. Antibacterial activity

**Fig. 9:**
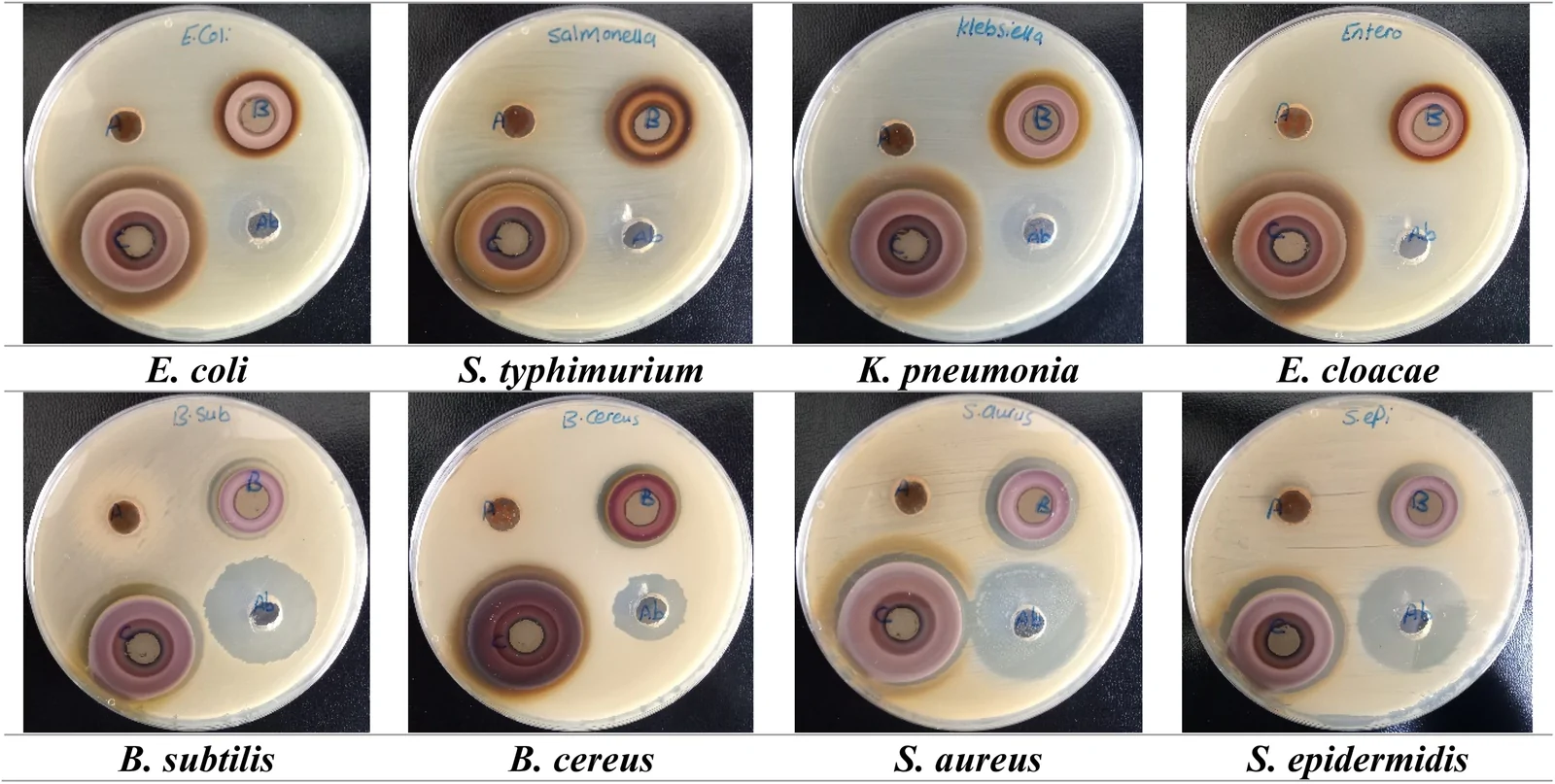
Representative Petri dish images illustrating the antibacterial activity of cinnamon extract, Ag/ZnO NC, Ag/ZnO/CMC NC, and azithromycin (positive control, 1 mg mL^−1^) against various pathogenic bacteria using the agar well diffusion assay. The wells in each plate were designated as follows: A = cinnamon extract, B = Ag/ZnO NC, C = Ag/ZnO/CMC NC, and Ab = azithromycin. Clear inhibition zones surrounding the wells (measured in millimeters) indicate effective inhibition of bacterial growth, whereas the absence of inhibition zones indicates no detectable antibacterial activity.

The antibacterial activities of cinnamon extract, Ag–Zn NC, and Ag–Zn-CMC NC against different types of Gram-positive and Gram-negative pathogenic bacteria were investigated using the agar well diffusion method, and results are presented in Table S5 and Fig. 9. The cinnamon extract had no antibacterial activities against any type of tested bacteria, indicating that the bioactive components of the cinnamon extract were not active enough to suppress the bacterial growth at the used concentration. Conversely, the antibacterial activities of Ag–Zn NC and Ag–Zn-CMC NC against the examined bacteria were highly significant.

Among the materials that were examined, the Ag–Zn-CMC NC material exhibited strong antibacterial properties, showing a significant increase in size of the inhibition zone (*p* < 0.05) in comparison with Ag–Zn NC and azithromycin in most cases. Inhibition zones produced by the Ag–Zn–CMC NC material against Gram-negative bacteria ranged between 30.0 ± 0.8 mm and 32.0 ± 1.1 mm. On the other hand, the Ag–Zn NC material showed comparatively smaller inhibition zones in the range of 21.0 ± 0.5 mm and 22.0 ± 0.8 mm. The maximum antibacterial property of the Ag–Zn-CMC NC material against Gram-negative bacteria was recorded against *S. typhimurium* and *K. pneumoniae* bacteria, which showed inhibition zones of 32.0 mm. Interestingly, the Ag–Zn-CMC NC displayed superior activity compared with azithromycin against *E. coli*, *S. typhimurium*, and *K. pneumoniae*.

Similar results were obtained for the antibacterial activity against Gram-positive bacterial strains. Ag–Zn-CMC NCs displayed inhibition zones varying between 31.0 ± 1.1 mm and 36.0 ± 1.2 mm, whereas Ag–Zn NCs exhibited inhibition zones from 21.0 ± 0.6 mm to 23.0 ± 0.8 mm. Maximum antibacterial efficacy was found for *S. aureus* bacteria, as Ag–Zn-CMC NCs produced the inhibition zone of 36.0 ± 1.2 mm, which is more than that of the azithromycin antibiotic, which was equal to 32.0 ± 1.0 mm. These findings indicate that the incorporation of carboxymethyl cellulose substantially enhanced the antibacterial performance of the Ag–Zn nanocomposites.

The high efficacy of the Ag–Zn-CMC NC can be explained by the synergy between the Ag nanoparticles, zinc compounds, and the CMC polymer. The protective effect that can be provided by the CMC coating can help achieve better stability and uniform distribution of the particles in the medium, as well as more efficient interaction with bacteria. Moreover, due to their size, nanocomposites can easily enter bacterial cells and damage their structure and metabolism.

The results acquired during the current study correlate with other previously received data on Ag/ZnO-CMC nanocomposites. Nanotechnology Lungu *et al.* showed excellent antimicrobial efficiency of CMC-capped Ag–ZnO nanocomposites for multiple pathogenic bacterial strains, due to the increased nanoparticle stability and dispersion in the CMC matrix.^33^ Furthermore, according to Khamidov *et al.*, sodium carboxymethyl cellulose nanocomposites with biologically synthesized Ag–ZnO hybrid fillers had a high biological efficiency due to their synergistic interaction.^42^ As mentioned by Salem *et al.*, carboxymethyl cellulose-stabilized Ag nanocomposites were effective against microorganisms due to enhanced bioavailability of nanoparticles provided by the polymer matrix.^43^ Additionally, Alahmadi and Hussein showed the increased biological performance of Ag/ZnO nanocomposites, which is caused by an elevated ROS production and cellular membrane destruction.^37^ Finally, as suggested by Bayisa *et al.*, ZnO–Ag nanocomposites displayed persistent and wide-spectrum antimicrobial properties as a result of continuous Ag^+^ ion release and oxidative stress formation.^44^

Further investigations on the antibacterial efficacy of the Ag/ZnO/CMC NC were carried out *via* the broth microdilution test. The OD_600_ results showed the dose-dependent inhibition effect on the bacterial growth (Table S6). The most potent inhibition effect was exerted by the NC on *B. subtilis* with the MIC value of 3.125 µg mL^−1^, then on *K. pneumoniae* and *S. aureus* with the MIC values of 6.25 µg mL^−1^ each; the MIC value for *S. typhimurium* was relatively high, which reached 12.5 µg mL^−1^. At concentrations above the respective MIC values, OD_600_ values remained close to the background level, indicating effective suppression of bacterial proliferation, while progressively higher OD_600_ values were observed at sub-MIC concentrations. The higher sensitivity of *B. subtilis* than Gram-negative bacteria could be linked to differences in the structure of cell envelope and interaction of the Ag/ZnO/CMC surface with bacteria. The observed antibacterial effect is caused by the effects of both Ag and ZnO, including disruption of bacterial membranes, oxidative stress because of the formation of ROS, and interference with vital bacterial functions. Thus, the results of MIC confirm the broad-spectrum antibacterial activity of the Ag/ZnO/CMC NC against both Gram-positive and Gram-negative bacteria.

Antimicrobial activity of the Ag/ZnO/CMC nanocomposite could be associated with a combination of such factors as metal ion release, ROS production, and interaction with microbial cells. In particular, interaction of the Ag nanoparticles with the bacterial cell surface can lead to destabilization of the membrane due to electrostatic attraction, whereas Ag^+^ ions will act on thiol-containing molecules of the bacterial cells. Moreover, the Zn-containing fraction could increase ROS production *via* the formation of ˙OH and O_2_˙^−^ radicals. The above-mentioned mechanisms could explain why there is an increased antimicrobial activity for bimetallic Zn–Ag nanocomposites and Ag–Fe–Zn nanocomposites^45,46^ and Ag/ZnO nanocomposites.^47^ It should be mentioned that the role of the CMC matrix is also essential because of enhanced dispersion of metal nanoparticles and prolonged interaction of nanoparticles with the environment.^48^

It is also worth mentioning that the results obtained in the radical scavenging experiments directly confirm the participation of ROS in the photocatalytic activity of Ag/ZnO/CMC NC. The presence of IPA led to a significant decrease in the degradation of CV from 99.33 ± 1.00% to 58.42 ± 2.15%, which corresponds to the inhibition of 41.20%, while the BQ inhibited CV degradation to 69.76 ± 1.84%, which corresponds to the inhibition of 29.84%. These data prove the participation of ˙OH and O_2_˙^−^ radicals, respectively. However, inhibition caused by the addition of EDTA-2Na and NaN_3_ was lower (17.28% and 7.91%), which shows comparatively low contribution of h^+^ and ^1^O_2_, correspondingly.

The metal leaching data offer further evidence regarding the antimicrobial and photocatalytic characteristics of the material. While the actual concentrations of the released metal ions have increased with the amount of the catalyst, the proportions of metal leaching were still relatively small. At the highest catalyst concentration (2.00 g L^−1^), the concentration of Ag^+^ and Zn^2+^ was 0.53 ± 0.11 and 1.29 ± 0.09 mg L^−1^, respectively, which corresponded to only 0.13 ± 0.01% and 0.32 ± 0.02% of the total Ag and Zn amounts. Therefore, even though released Ag^+^ and Zn^2+^ could play a role in the antimicrobial activity of the material, the extremely low percentage of the release means that uncontrollable dissolution of metals cannot be the main mechanism of action. Rather, the antibacterial effect of the catalyst can be explained by the combination of direct contact of nanoparticles with bacteria, their oxidative stress, and membrane damage. Similar synergistic effects of metal ions, ROS formation, and membrane/biofilm damage have been reported for AgCu nanocomposite.^49^

In conclusion, all the above results suggest the antimicrobial property of Ag/ZnO/CMC NC to be multimodal, where Ag and Zn play complementary roles of antimicrobials while CMC matrix helps in dispersion and stability of the active nanostructure. The high role of ˙OH and O_2_˙^−^ radical anions, identified *via* scavenger experiments, and low release of Ag^+^/Zn^2+^ ions show the ability of the nanocomposite to have a high level of antimicrobial and photocatalytic activity without the need for the dissolution of its metal component. Such properties of Ag/ZnO/CMC NC make it promising for environmental antimicrobial and photocatalytic applications.^45–48^

### 3.4. Crystal violet degradation

#### 3.4.1. Effect of operational parameters on photocatalytic degradation

**Fig. 10:**
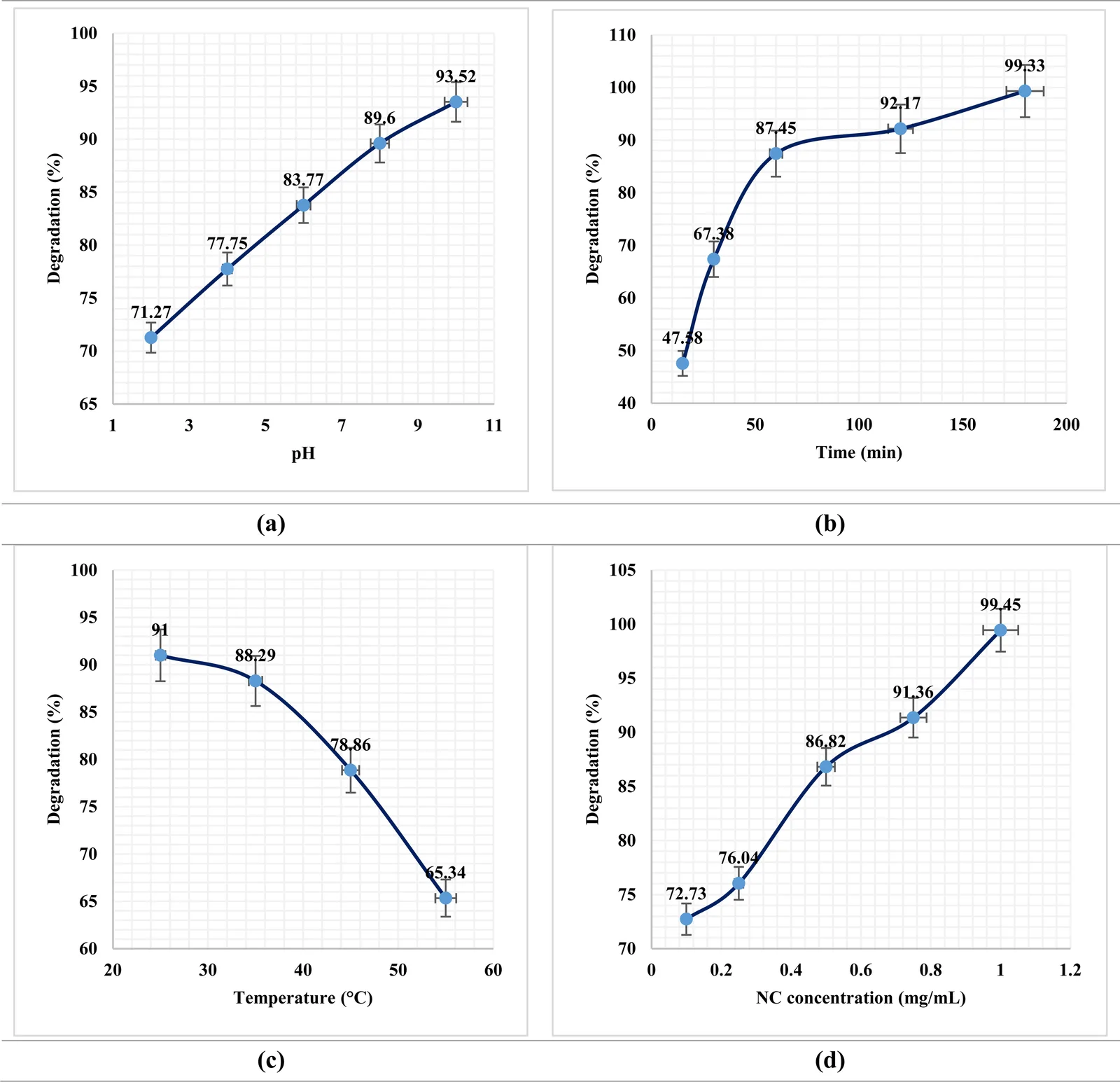
(a) Effect of pH on the degradation efficiency of CV by Ag/ZnO/CMC nanocomposites through the process of photocatalytic degradation, where the performance is observed to increase at high pH levels owing to high levels of ˙OH radicals produced. (b) Degradation efficiency of CV with respect to time to study the rate of the photocatalytic degradation process of CV, noting that it takes place rapidly initially, followed by degradation through photooxidation. (c) Effect of catalyst concentration on the efficiency of degradation. (d) Effect of temperature on the degradation performance of CV.

The degradation efficiency of Crystal violet (CV) by the as-prepared Ag/ZnO/CMC NC *via* photocatalysis was examined under varying conditions, such as pH value, irradiation time, catalyst concentration, temperature, and salt concentration. The outcomes are presented in Table S7 and shown in Fig. 10.

The impact of pH on the rate of CV degradation is quite evident from Fig. 10a. The degradation percent steadily rose from 71.27 ± 2.10% at pH 2 to 93.52 ± 1.50% at pH 10. Photocatalytic activity under alkaline conditions is mainly due to increased hydroxyl radical (˙OH) formation. Hydroxyl radical is regarded as the principal oxidant for dye breakdown. Under alkaline conditions, high OH^−^ concentration results in greater availability of these radicals on the surface of the catalyst, hence speeding up the oxidation process.^50,51^ Likewise, the negatively charged catalyst surface in an alkaline environment could facilitate better electrostatic interaction between the catalyst and positively charged dye molecules.^52^

The influence of irradiation time on the degradation of CV is illustrated in Fig. 10b. It was observed that the efficiency of degradation had an abrupt increase from 47.58 ± 2.50% in 15 minutes to 99.33 ± 1.00% in 180 minutes. The rapid degradation in the initial stage of the process can be attributed mainly to the presence of many active sites and effective adsorption of the dye molecules by the catalyst. However, when the degradation progresses, the photocatalytic oxidation process takes place prominently, leading to the production of reactive oxygen species such as ˙OH and O_2_˙^−^ radicals continuously. Similar degradation behavior was found for other Ag–ZnO nanocomposites and CMC-based photocatalysts.^53,54^

The quantity of catalyst used was another significant factor that contributed to the photodegradation process (Fig. 10c). An increment in the concentration of the nanocomposite from 0.10 to 1.00 mg mL^−1^ caused an increase in the percentage of photodegradation from 72.73 ± 2.30% to 99.45 ± 0.90%. This increase could be attributed to the presence of an increased active surface area and increased photocatalytic sites for ROS formation. Excessive catalyst concentration may lead to decreased effectiveness of the photodegradation process due to particle aggregation and light scattering, as reported earlier.^54,55^

The effect of temperature on degradation efficiency was demonstrated in Fig. 10d. With the rise of temperature from 25 °C to 55 °C, the degradation efficiency declined from 91.00 ± 1.20% to 65.34 ± 2.10%, implying that increased temperatures had an adverse impact on the equilibrium of the adsorption–photocatalysis process. The decline in degradation efficiency due to higher temperatures may be related to the reduced CV affinity for the catalyst surface and faster electron–hole recombination rates.^52^

Additionally, the presence of salt has considerably influenced the rate of degradation through photocatalysis. The increase in ionic strength by adding CaCl_2_ caused the rate of degradation to decrease from 99.28 ± 0.90% to 76.16 ± 2.10% (Table S7). This can be due to the competitive adsorption of dye molecules and calcium ions and the scavenging of reactive species by salt ions.^55^

The results shown in Table S8 also support the synergistic effect of the components. While the blank and CMC resulted in 5.8 ± 0.74% and 10.6 ± 1.18% degradation, respectively, Ag/CMC and ZnO/CMC recorded 74.8 ± 1.41% and 67.5 ± 1.52% degradation, respectively. In comparison, the combination of Ag and ZnO/CMC led to an enhanced degradation of 84.7 ± 1.33%, while the combined Ag/ZnO/CMC NC gave rise to a much higher activity (99.33 ± 1.01%). Clearly, the significantly higher degradation activity of the nanocomposite in contrast to that of the physical mixture proves the synergistic effect resulting from the intimate combination of Ag, ZnO, and CMC, which can facilitate charge separation and improve the availability of reactive photocatalytic sites.

#### 3.4.2. Kinetic modeling of crystal violet degradation

**Fig. 11:**
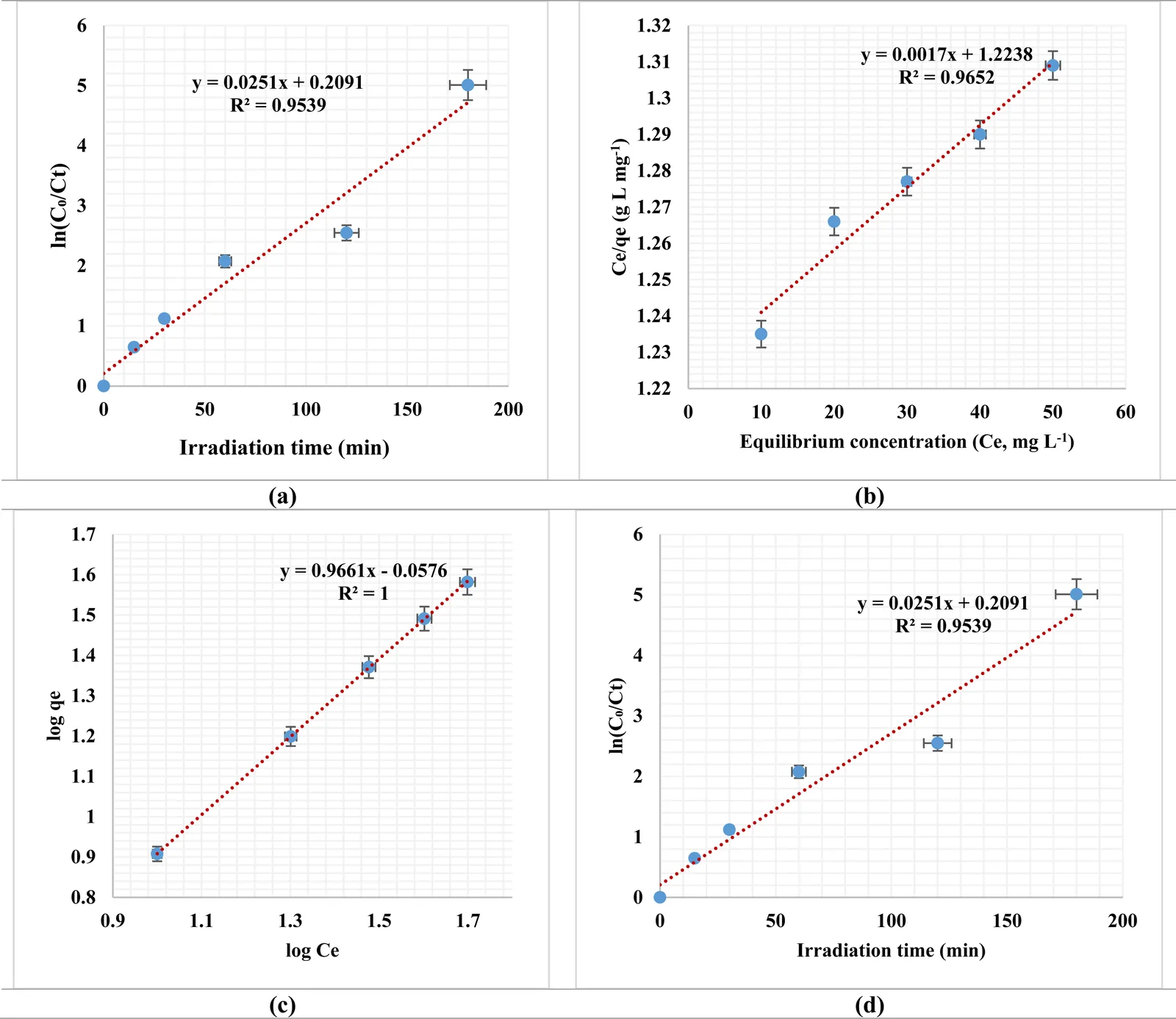
(a) Pseudo-first-order kinetic plot for the photocatalytic degradation of Crystal violet (CV) using Ag/ZnO/CMC nanocomposite, showing a linear relationship between ln(*C*_0_/*C*_*t*_) and irradiation time. (b) Langmuir adsorption isotherm plot for Crystal violet adsorption onto Ag/ZnO/CMC nanocomposite. (c) Freundlich adsorption isotherm for Crystal violet adsorption on Ag/ZnO/CMC nanocomposite, demonstrating heterogeneous surface adsorption. (d) Langmuir–Hinshelwood kinetic plot of Crystal violet over Ag/ZnO/CMC nanocomposite, exhibiting pseudo-first-order behavior with an apparent rate constant.

Kinetics studies of the photocatalytic degradation of CV by Ag/ZnO/CMC NC (Tables S9–S12 & Fig. 11a–d) were carried out using both the pseudo-first-order (PFO) and Langmuir–Hinshelwood (L–H) kinetic models. The values of the kinetic parameters are provided in Tables S9 and S12, respectively, while Fig. 11a and d represent their plots. The linear dependency of the ln(*C*_0_/*C*_*t*_) *vs.* irradiation time demonstrated the pseudo-first-order behavior of the reaction process (Fig. 11a). The rate constant (*k*) value of 0.0251 min^−1^ with *R*^2^ = 0.9539 confirms the good agreement between the experimental data and the kinetic model. The half-life value (*t*_1/2_) was estimated as 24.2 min.

Likewise, the Langmuir–Hinshelwood model demonstrated good applicability in terms of its adequacy to the described degradation process (see Fig. 11d). Specifically, the apparent rate constant (*k*_app_) equaled 0.0286 ± 0.0012 min^−1^, while the adsorption constant (*K*_ads_) amounted to 0.082 ± 0.004 mg L^−1^. It means that, according to the adequacy of the L–H approach used, the adsorption of CV on the surface of the catalyst is a key stage preceding the process of photocatalytic oxidation.

As shown in Table 1, the comparison of the kinetics of the models indicated that the Langmuir–Hinshelwood mechanism had the highest value of *R*^2^ (*R*^2^ = 0.972), while the pseudo-first-order mechanism had the second highest value (*R*^2^ = 0.968). The pseudo-second-order kinetic model showed less relevance (*R*^2^ = 0.941). This clearly supports the assertion that the photodegradation process is mainly controlled by the oxidation reaction at the surface of the material and not only through chemisorption processes.

**Table 1:** Kinetic model comparison for Crystal violet degradation using Ag/ZnO/CMC NC

Model	Equation	Rate constant	*R* ^2^	Interpretation
Pseudo-first-order	ln(*C*_0_/*C*_*t*_) = *kt*	0.0286 ± 0.0012 min^−1^	0.968	Best fit
Pseudo-second-order	*t*/*q*_*t*_ = 1/*k*_2_*q*_e_^2^ + *t*/*q*_e_	0.00142 ± 0.00008 g mg^−1^ min^−1^	0.941	Less suitable
Langmuir–Hinshelwood	*r* = *kKC*/(1 + *KC*)	0.0312 ± 0.0015 min^−1^	0.972	Highly applicable

#### 3.4.3. Adsorption isotherm analysis

An adsorption isotherm study was carried out for a better understanding of the interactions between CV and Ag/ZnO/CMC NC surface. Analysis of the adsorption was done by two models: the Langmuir and the Freundlich isotherm models, and are discussed below in Tables S10, S11, and Fig. 11b and c.

From the Langmuir isotherm model, the linearity of the *R*^2^ value is quite high (*R*^2^ = 0.9652). The linear relationship shows that adsorption occurs by the monolayer adsorption of CV molecules on the homogeneous surface of the catalytic material. From the model, *Q*_max_ was obtained as 62.85 ± 1.42 mg g^−1^, and KL as 0.082 ± 0.004 L mg^−1^. The favorable adsorption behavior signifies the affinity of the nanocomposite towards cationic dye molecules. The effect of CMC in the system might be due to hydrogen bonding and electrostatic interaction with the dye.

Similarly, the Freundlich isotherm model was also found to be in excellent agreement with the experimental data (*R*^2^ = 1.0), revealing the heterogeneous nature of the adsorption process (Fig. 11c). Freundlich constant KF was 5.74 ± 0.18, while the adsorption intensity constant (*n* = 2.41 ± 0.09) indicated the favorable nature of the adsorption process. The value of *n* is higher than unity, indicating the highly favorable nature of the adsorption process and the spontaneity of the adsorption process.^53^ This indicates that the nanocomposite of Ag/ZnO/CMC has both homogeneous and heterogeneous nature of adsorption sites due to its hybrid metallic-polymeric nature.

Degradation of Crystal violet (CV) dye through photocatalysis employing Ag/ZnO/CMC NC adhered to the kinetics of the Langmuir–Hinshelwood equation based on its first-order reaction model, as shown in Table S12 & Fig. 11d. The gradual rise of the ln(*C*_0_/*C*_*t*_) value from 0.646 to 5.010 within 15 to 180 minutes correlated well with the marked improvement in degradation efficacy from 47.58% to 99.33%, illustrating the efficient photocatalytic response of the prepared nanocomposite in visible light. A high correlation coefficient (*R*^2^ = 0.9539) was achieved during the kinetic fitting, implying an agreement between experimental results and the applied kinetic equation. Apparent rate constant (*k*_app_) value was determined to be equal to 0.0286 ± 0.0012 min^−1^ while the half-life (*t*_1/2_) was found to be equal to 24.2 min, which indicated rapid kinetics of the degradation process and high efficiency of catalyst activity. In addition, the obtained value of the adsorption constant (*K*_ads_ = 0.082 ± 0.004 L mg^−1^) indicated good interaction between Crystal violet molecules and active sites of the Ag/ZnO/CMC NC. The high model validity confirmed that the degradation process was strongly governed by surface adsorption and photocatalytic reaction mechanisms occurring simultaneously on the nanocomposite surface.

#### 3.4.4. Thermodynamic investigation

**Fig. 12:**
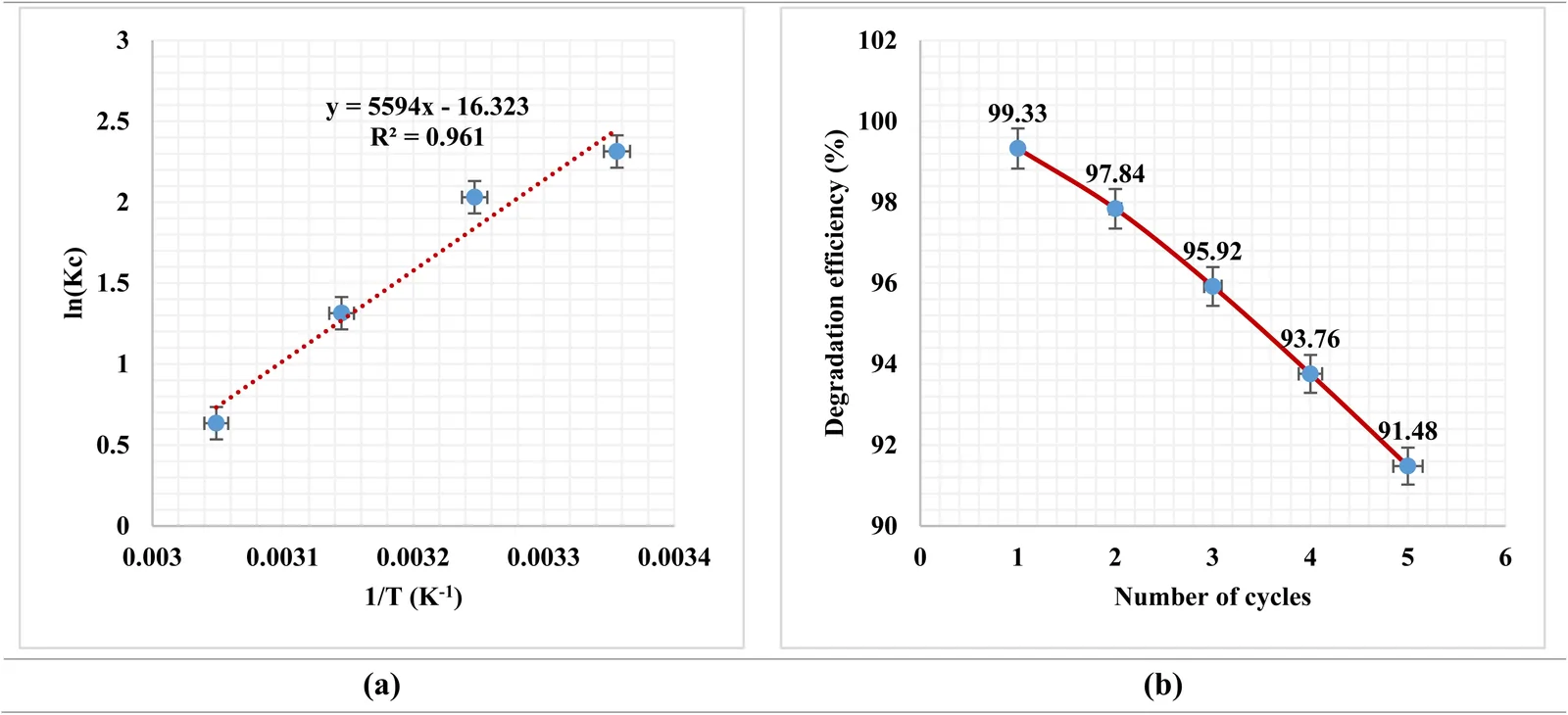
(a) van't Hoff plot for thermodynamic analysis of Crystal violet degradation using Ag/ZnO/CMC nanocomposite, confirming endothermic and spontaneous behavior. (b) Reusability performance of Ag/ZnO/CMC nanocomposite for repeated photocatalytic degradation of Crystal violet over five cycles.

The thermodynamic parameters of the degradation of CV have been estimated using van't Hoff analysis, and these values are listed in Table 2 and Fig. 12a below. The positive Δ*H*° value (+46.65 ± 1.15 kJ mol^−1^) shows that the process of photocatalytic degradation is an endothermic one. On the other hand, the positive Δ*S*° value (+172.3 ± 4.8 J mol^−1^ K^−1^) signifies higher randomness at the solid–liquid interface.

Thermodynamic parameters for the photocatalytic degradation of Crystal violet using Ag/ZnO/CMC NC calculated from van't Hoff analysis

**Table:** 

Temperature (°C)	Temperature (K)	Degradation (%)	*K*_c_	ln(*K*_c_)	1/*T* (K^−1^)
25	298	91.00 ± 1.20	10.111 ± 0.35	2.314 ± 0.028	0.003356
35	308	88.29 ± 1.30	7.621 ± 0.30	2.031 ± 0.026	0.003247
45	318	78.86 ± 1.60	3.724 ± 0.18	1.315 ± 0.021	0.003145
55	328	65.34 ± 2.10	1.887 ± 0.12	0.635 ± 0.018	0.003049

**Table:** 

Parameter	Value
Slope	−5594
Intercept	16.323
*R* ^2^	0.961
Δ*H*° (kJ mol^−1^)	+46.65 ± 1.15
Δ*S*° (J mol^−1^ K^−1^)	+172.3 ± 4.8
Δ*G*° (298 K) (kJ mol^−1^)	−4.73 ± 0.22
Reaction nature	Spontaneous and endothermic

The spontaneity of the reaction has been proven by the negative Gibbs free energy (at 298 K; Δ*G*° = −4.73 ± 0.22 kJ mol^−1^). The van't Hoff plot has been found to be linear (*R*^2^ = 0.961), which further supports the thermodynamically consistent degradation process. Spontaneous and endothermic behaviors of photocatalytic degradation have been noted for similar systems before.^52,56^

#### 3.4.5. Reusability and stability of Ag/ZnO/CMC NC

Recyclability and stability of Ag/ZnO/CMC NCs were tested during five degradation cycles (Table S13 and Fig. 12b). Degradation rate was slightly reduced from 99.33 ± 1.00% in the first cycle to 91.48 ± 1.52% in the fifth cycle; meanwhile, residual activity exceeded 92%. This drop in the efficiency of the photo-catalyst can be caused by fouling of its surface, loss of a part of the catalyst during the recovery process, and buildup of the intermediates formed during the degradation process on the surface of the photo-catalyst. However, despite this drop in the catalytic efficiency, the nanocomposite exhibited high stability after multiple uses. This indicates that this nanocomposite is highly stable and resistant to photocorrosion.^53,56^ This can be associated with the protective effect of the CMC matrix.

#### 3.4.6. Photocatalytic performance in different water matrices

The application of Ag/ZnO/CMC NCs in real-life conditions was tested *via* different aqueous media, including distilled water, tap water, and seawater (Table 3). Distilled water showed the most significant efficiency in degradation (99.33 ± 1.00%), followed by tap water (93.84 ± 1.28%) and seawater (85.67 ± 1.74%).

**Table 3:** Reversibility and photocatalytic degradation performance of Ag/ZnO/CMC NC for Crystal violet removal using different water sources under visible light irradiation. Data are expressed as mean ± SD (*n* = 3). The nanocomposite retained considerable photocatalytic activity in complex aqueous environments, demonstrating good environmental applicability and operational stability

Water source	Initial dye concentration (mg L^−1^)	Degradation efficiency (%)	Residual activity (%)	Observation
Distilled water	50	99.33 ± 1.00	100.0	Highest degradation efficiency due to the absence of interfering ions
Tap water	50	93.84 ± 1.28	94.5	Slight reduction attributed to dissolved minerals and ions
Sea water	50	85.67 ± 1.74	86.2	Lower degradation efficiency due to high salinity and ionic competition

The lower efficiency in seawater can be explained by high salinity and interference from other ions like Cl^−^ and SO_4_^2−^. Such ions negatively affect the activity of ROS species and inhibit their interaction with the dye molecules. Nonetheless, the catalyst still showed sufficient catalytic activity in complex water media, indicating possible applications for wastewater treatment.^55^

#### 3.4.7. Comparative photocatalytic performance

The photodegradation activity of the Ag/ZnO/CMC nanocomposite synthesized was compared with other photocatalyst materials used for the degradation of CV and listed in Table 4. The Ag/ZnO/CMC NC showed excellent photocatalytic activity, with more than 99.33 ± 1.00% removal of the dye within 180 minutes of visible light exposure. The increased efficiency might be due to the synergistic effect of silver nanoparticles, zinc oxide, and CMC polymer matrix in the nanocomposite. In silver nanoparticles, electrons work as sinks by preventing electron–hole recombination to enhance charge separation. Furthermore, the CMC matrix enhances the dispersion of nanoparticles, surface stability, and adsorption of dye molecules, which helps mass transfer and catalytic processes. Such synergy effect mechanisms have been studied earlier in metal oxides on polymer-based photocatalysts.^53–56^ In comparison to other existing photocatalysts using ZnO and Ag in the literature, the current Ag/ZnO/CMC NC exhibited better degradation ability at relatively mild visible light irradiation, proving itself as a good candidate photocatalyst for wastewater treatment applications.

**Table 4:** Comparative photocatalytic performance of Ag/ZnO/CMC NC with literature reports

Catalyst	Dye	Experimental conditions	Degradation (%)	Reference
Ag/ZnO/CMC NC	Crystal violet	Visible light, 180 min	99.33 ± 1.00	This work
Cloisite 30B/ZnO/Ag_2_O NC	Crystal violet	Sono-photocatalysis, 120 min	*ca.* 92 ± 2.00	50
ZnO nanoparticles	Crystal violet	Visible light, 180 min	*ca.* 80 ± 3.00	51
Nanochitosan/CMC/TiO_2_ biocomposite	Crystal violet	Visible light irradiation	*ca.* 96 ± 1.50	53
Fe_3_O_4_/CMC@Cysteine-ZnO NC	Methyl green	Visible light irradiation	*ca.* 93 ± 1.80	54

#### 3.4.8. FTIR analysis and proposed mechanism of crystal violet degradation

**Fig. 13:**
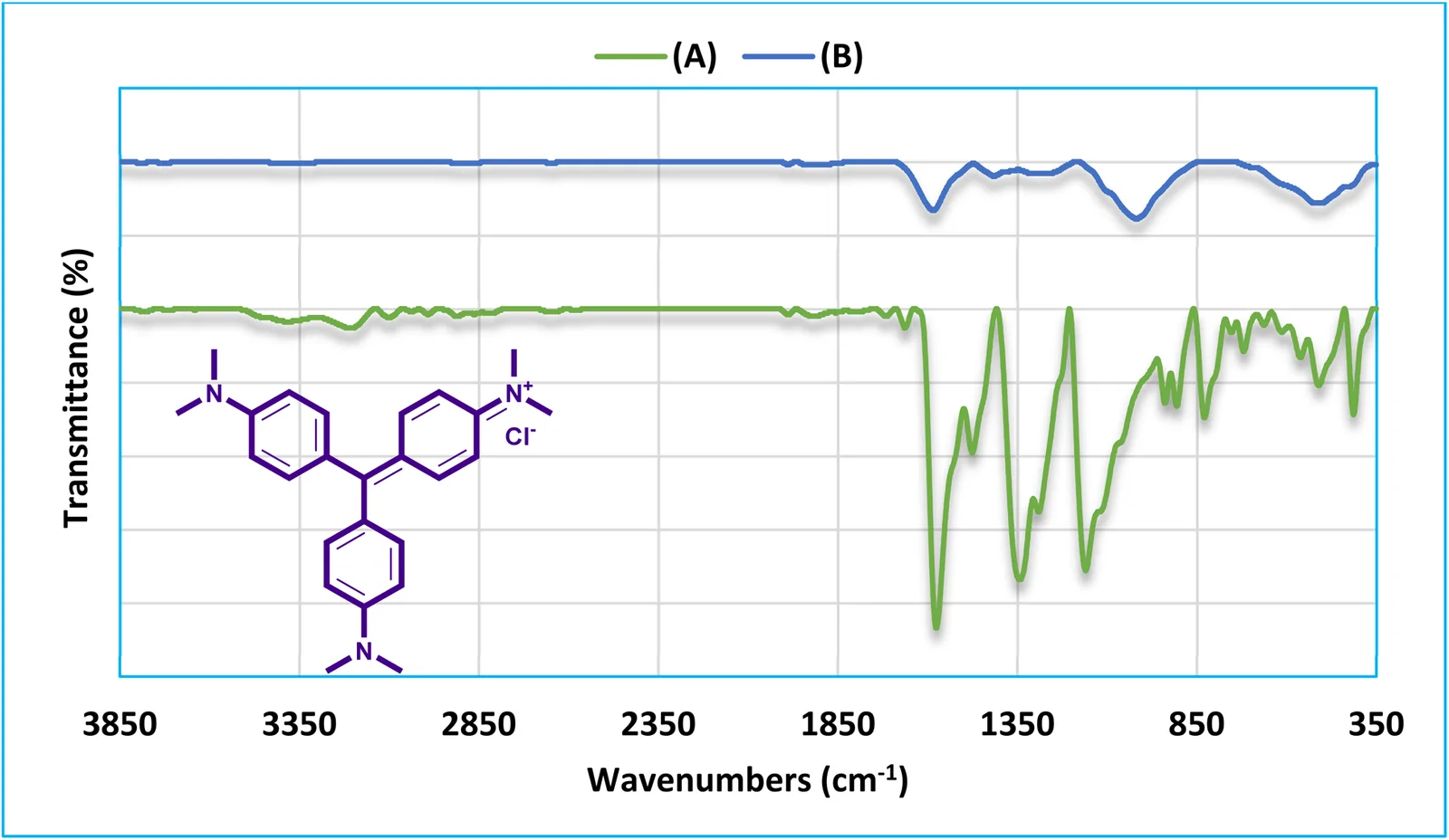
FTIR spectra of (A) Crystal violet and (B) Crystal violet treated with Ag/Cu/CMC NC.

FTIR analysis of pristine Crystal violet (CV) dye and CV dye exposed to Ag/ZnO/CMC NC is shown in Fig. 13 & Table S14. FTIR spectrum of pristine CV showed several characteristic absorption bands of the structure of triphenylmethane dye. The broad peaks in the region of 3380 and 3190 cm^−1^ were related to O–H and N–H stretching vibrations resulting from water adsorption and amine groups, respectively. The strong peak observed at 1660 cm^−1^ was due to C=N stretching vibration, whereas peaks in the region of 1580 and 1520 cm^−1^ were indicative of C=C stretching vibration of aromatic rings. Additionally, the bands detected between 1480 and 1290 cm^−1^ were assigned to be associated with the stretching vibration of the C–N bond and aromatic skeletal vibration of the triphenylmethane system. Meanwhile, the bands recorded at 1220, 1160, 1110, and 1060 cm^−1^ were assigned to the stretching vibration of C–N and C–C bonds, whereas those recorded below 1000 cm^−1^ were due to the bending vibration of aromatic C–H and deformation of substituted benzene rings. These findings can be correlated to the FTIR spectra reported for crystal violet and its photocatalytic degradation reaction.^57,58^

Following exposure to the Ag/ZnO/CMC NC, significant spectral differences were noted, confirming the degradation and structural transformation of the CV molecules. The loss of the strong peak at 1660 cm^−1^ confirmed that the C=N chromophore had either been broken or oxidized, which is responsible for the violet coloration of the dye. In addition, the decrease in the intensity and disappearance of various aromatic skeleton vibrations in the 1520–1220 cm^−1^ region also implied that the triphenylmethane aromatic skeleton had been degraded and the dimethylamino auxochromes destroyed. The presence of faint peaks at 1580 and 1420 cm^−1^ might be due to the presence of some aromatic intermediates formed during partial oxidation.

The presence and appearance of a band at 3350 cm^−1^ following treatment signified the development of intermediate compounds that contain hydroxyl groups and adsorbed water molecules; the latter can be produced as a result of the reactions of oxidation–hydroxylation. In addition, the development of bands at 1020 and 990 cm^−1^ signified the production of intermediate compounds that are oxygenated with small molecular size, such as alcohols, aldehydes, and carboxyl groups, following the ring–opening reactions.

This degradation mechanism is believed to be a consequence of the cooperative photoreactivity between the nanoparticles of Ag and Zn particles incorporated into the CMC matrix. The absorption of photons by the Ag/ZnO/CMC NC triggers the promotion of electrons from the valence band to the conduction band, creating the formation of electron–hole pairs. The photo-generated electrons then participate in redox reactions with the oxygen molecules present in the solution, producing superoxide radicals (O_2_˙^−^), while the photo-generated holes undergo redox reactions with water molecules/hydroxide ions, forming hydroxyl radicals (˙OH). Reactive oxygen species initiate the oxidation reactions that affect the chromophoric structure of Crystal violet by undergoing *N*-demethylation, breaking the carbon bridge at the center, and eventually destroying the aromatic ring system. This is reflected in the continuous disappearance and weakening of bands related to the aromatic amine and conjugated functional groups in FTIR spectra.

**Fig. 14:**
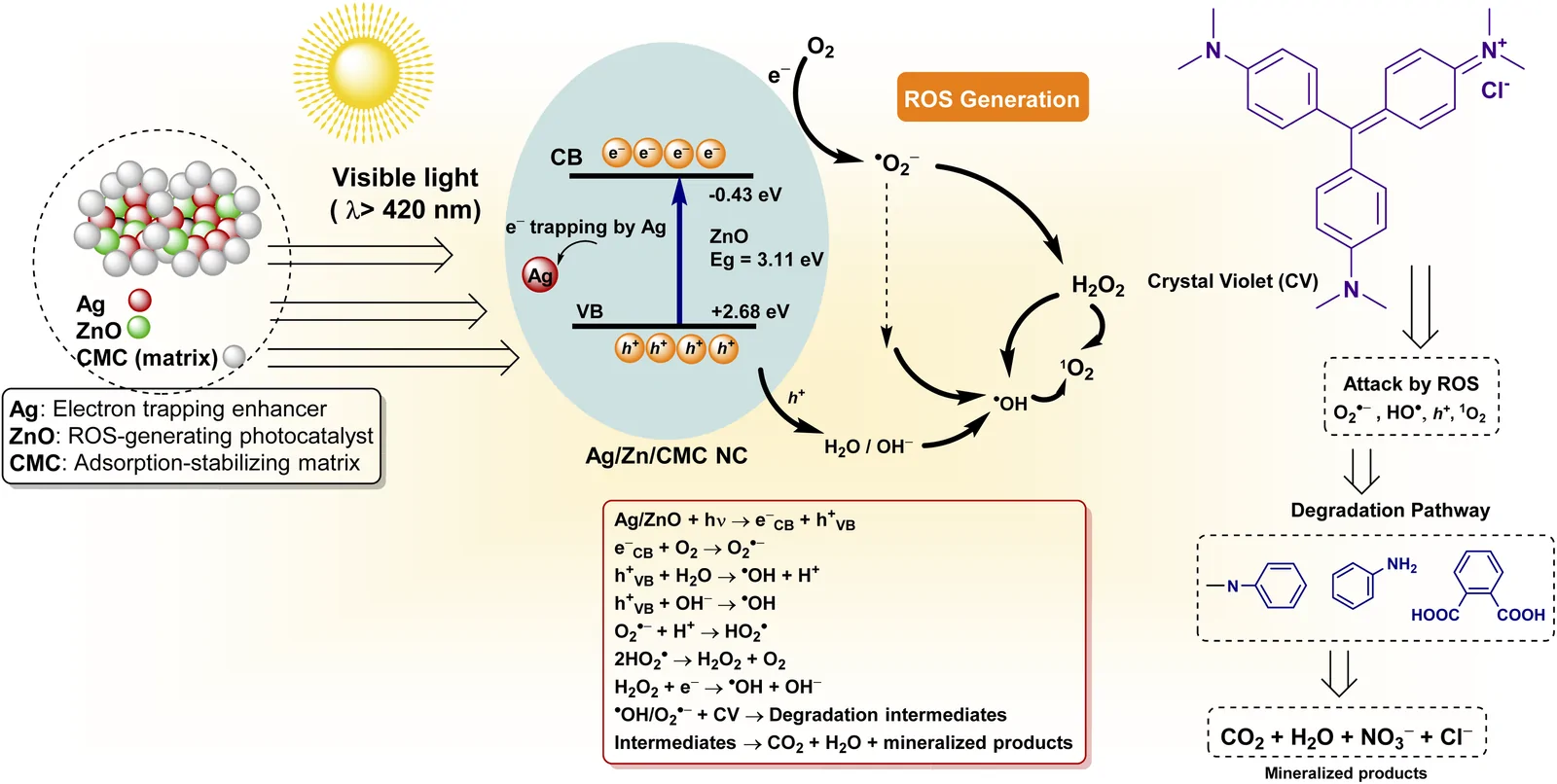
Proposed photocatalytic mechanism and ROS pathway.

The incorporation of Ag nanoparticles must have played an important role in the absorption of light and the conduction of electrons without allowing the recombination of both electrons and holes. However, the importance of the zinc salt, such as ZnO, could not be ignored during the synthesis of the reactive oxygen species *via* redox reactions. Additionally, the CMC polymer matrix offered several sites for the adsorption of Crystal violet molecules. Therefore, the degradation could have possibly occurred *via* a synergy between adsorption and photocatalytic oxidation pathways, resulting in intermediate molecules being formed and then partial mineralization of such compounds to CO_2_, H_2_O, and inorganic salts. This degradation pathway agrees well with other works that have focused on the degradation of Crystal violet using Ag/ZnO NC, wherein hydroxyl (˙OH) and superoxide radicals (O_2_˙^−^) were recognized as the main active species responsible for breaking down chromophores and aromatic rings.^57,58^ Carboxymethyl cellulose (CMC) increased Crystal violet degradation as it acted as a stabilization and absorption support matrix for Ag/ZnO nanoparticles. Hydroxyl and carboxyl groups of CMC increased dye adsorption and provided better dispersion of nanoparticles, thereby improving the interaction of the reactive oxygen species with dye molecules. Such results were also observed by Sugashini *et al.*^53^ in their studies on enhanced photocatalytic degradation under visible light using CMC-based biocomposites due to improved adsorption and charge separation. These FTIR observations support the proposed photocatalytic degradation pathway presented in Fig. 14.

#### 3.4.9. Metal ion release

The leaching behavior of Ag^+^ and Zn^2+^ from Ag/ZnO/CMC NC was studied using catalyst concentrations ranging between 0.25 and 2.00 g L^−1^ and these findings are summarized in Table S15. The concentrations of Ag^+^ and Zn^2+^ in the reaction medium both increased gradually with an increase in the catalyst concentration. For instance, the leaching of Ag^+^ ions was found to increase from 0.08 ± 0.01 mg L^−1^ at a catalyst concentration of 0.25 g L^−1^ to 0.53 ± 0.11 mg L^−1^ at 2.00 g L^−1^ and that of Zn^2+^ increased from 0.21 ± 0.02 to 1.29 ± 0.09 mg L^−1^. This observation is expected due to the fact that a higher catalyst concentration leads to the availability of more Ag and Zn ions.

Even though the amount of metals increased, the percentage of the total metal which was released reduced as the catalyst dose increased. The amount of Ag that was released reduced from 0.16 ± 0.02% at 0.25 g L^−1^ to 0.13 ± 0.01% at 2.00 g L^−1^ while Zn decreased from 0.42 ± 0.04% to 0.32 ± 0.02%. Less than 0.5% of the total Ag and Zn was therefore released even at the lowest catalyst dose studied. The low percentages suggest that the metals are well immobilized in the CMC-based nanostructure, demonstrating good resistance toward dissolution under the photocatalytic conditions.

The higher release of Zn^2+^ compared to Ag^+^ could be due to the fact that the Zn containing species are easily oxidatively dissolved in the aqueous photocatalytic system while Ag remains more strongly adsorbed on the Ag/ZnO/CMC composite. Significantly, the very small total metal release indicates that the CV degradation cannot be attributed mainly to uncontrollable dissolution of either of these metals. Instead, this indicates that the heterogenous Ag/ZnO/CMC NC plays the major part in the photocatalytic process.

This low metal ion leaching is important in practical terms for the regeneration and environmental applications of the catalyst. The leaching of too much silver or zinc can gradually change the composition of the catalyst and cause secondary metal pollution. In the present case, the low amount of leached ions found in relation to the concentration of the catalyst used proves the stability of the structure of the catalyst, thus, justifying its use as a relatively stable catalyst for CV decomposition. These findings provide additional evidence for the heterogeneous nature and stability of the Ag/ZnO/CMC NC during photocatalytic treatment.

#### 3.4.10. Identification of reactive species and photocatalytic degradation mechanism

For the determination of the major reactive species responsible for CV degradation through photocatalytic process, selective radical scavenging was performed with the aid of isopropanol (IPA), *p*-benzoquinone (BQ), EDTA-2Na, and sodium azide as scavengers of ˙OH, O_2_˙^−^, h^+^, and ^1^O_2_, respectively. These results are summarized in Table S16. In the absence of any scavenger, CV degradation by Ag/ZnO/CMC NC amounted to 99.33 ± 1.00%. Upon adding IPA, the degradation efficiency significantly decreased to 58.42 ± 2.15%, resulting in an inhibition of 41.20%. This clearly demonstrates the predominant involvement of hydroxyl radicals (˙OH) in CV degradation. Further, the degradation efficiency upon adding BQ decreased to 69.76 ± 1.84%, which is associated with an inhibition of 29.84%, with an inhibition of 29.84%, confirming the significant contribution of superoxide radicals (O_2_˙^−^). The effect of EDTA-2Na was lower in terms of the reduction of degradation down to 82.15 ± 1.63% (17.28% inhibition), indicating the secondary role of the photogenerated h^+^. Sodium azide showed insignificant inhibition of 7.91%, and CV degradation was still 91.47 ± 1.21%, suggesting that ^1^O_2_ has played an insignificant role in the reaction. Thus, based on the scavenging study, it could be concluded that ˙OH is the most significant reactive oxygen species followed by O_2_˙^−^ and h^+^, while ^1^O_2_ is the least significant one. According to the above scavenger study, the mechanism of the proposed photocatalytic reaction of Ag/ZnO/CMC NCs is presented in Fig. 14. Upon visible light irradiation, photo-induced electron–hole pair generation takes place, which leads to the separation of charges and the production of reactive oxygen species. The strong inhibition in the presence of IPA and BQ confirms the significant roles of ˙OH and O_2_˙^−^, respectively.

The suggested process of photocatalytic oxidation and the generation of ROS is shown in Fig. 14. After exposure to visible light, the Ag/ZnO semiconductor complex gets excited, producing electron–hole (e^−^/h^+^) pairs. Silver nanoparticles enhance the charge separation by capturing the electrons produced by photoexcitation.^59^ The excited electrons reduce the dissolved oxygen molecules to form superoxide radicals (O_2_˙^−^), whereas the generated photoholes oxidize the hydroxyl groups on the surface or from the water molecules to form hydroxyl radicals (˙OH). These reactive species then act on the chromophoric portion of the CV molecules, which results in oxidation, ring opening, the formation of intermediates, and finally mineralization to carbon dioxide (CO_2_), water (H_2_O), and inorganic compounds.^50,51,60^ Furthermore, the role of CMC lies in its ability to improve the performance of the photocatalytic process through better dispersion of nanoparticles, improved dye molecule adsorption, and stabilization of the catalyst sites. Future research should focus on complementary TOC and LC-MS analyses to verify the extent of CV mineralization and identify the intermediate degradation products generated during the photocatalytic process.

## 4 Conclusion

In this work, a new Ag/ZnO/CMC nanocomposites was greenly synthesized using cinnamon extract as a natural reducing and stabilizing agent. The integration of Ag and ZnO nanophases within the carboxymethyl cellulose (CMC) matrix produced a multifunctional nanocomposite with enhanced surface reactivity, antibacterial activity, and photocatalytic performance. Comprehensive characterization using UV-Vis spectroscopy, FTIR, XRD, SEM, TEM, EDS, XPS, BET/BJH analysis, and temperature-programmed desorption confirmed the successful formation of the hierarchical nanostructure, its mesoporous characteristics, strong interfacial interactions, and relatively uniform distribution of the inorganic nanophases within the CMC matrix.

In the present study, the Ag/ZnO/CMC NC achieved 99.33 ± 1.01% CV degradation after 180 min, substantially exceeding the activities of Ag/CMC (74.80 ± 1.41%), ZnO/CMC (67.50 ± 1.52%), and the corresponding physical mixture (84.70 ± 1.33%), thereby providing experimental evidence for enhanced performance following nanocomposite formation. The NC also exhibited broad-spectrum antibacterial activity, with MIC values of 3.125–12.5 mg mL^−1^, while Ag^+^ and Zn^2+^ release remained low (0.13 ± 0.01% and 0.32 ± 0.02%, respectively, at 2.00 g L^−1^), indicating good metal retention under the tested photocatalytic conditions. Radical-scavenging experiments further identified ˙OH as the predominant reactive species, followed by O_2_˙^−^ and h^+^, with inhibition values of 41.20%, 29.84%, and 17.28%, respectively, whereas ^1^O_2_ contributed only marginally (7.91% inhibition). These findings support the role of ROS-mediated oxidation in CV degradation and demonstrate the multifunctional potential of the Ag/ZnO/CMC NC for photocatalytic and antimicrobial applications.

The synthesized Ag/ZnO/CMC nanocomposite had high antibacterial potential against both Gram-positive and Gram-negative bacteria, with an inhibition zone up to 36.0 ± 1.2 mm for *S. aureus*. CMC coating of the nanoparticles improved stability and bacterial interaction and, hence, contributed to increased antibacterial properties compared to Ag/ZnO nanoparticles without CMC coating; at times, the antibacterial activity was even greater than the commercially produced drug Azithromycin. The Ag/ZnO/CMC NC exhibited pronounced antibacterial activity, with MIC values of 3.125–12.5 µg mL^−1^ against the tested Gram-positive and Gram-negative bacteria. In addition, the nanocomposite showed high photodegradation potential when subjected to visible light for the degradation of Crystal violet dye, whereby 99.33 ± 1.00% degradation took place within 180 min. The kinetics results showed that the photodegradation process followed the Langmuir–Hinshelwood mechanism, indicating the significance of enhanced adsorption of the photocatalyst. Increased photodegradation efficiency was attributed to effective charge separation, preventing charge carrier recombination by the use of silver nanoparticles, formation of reactive oxygen species by zinc oxide, and enhanced adsorption capacity of the CMC matrix.

The prepared nanocomposite showed high stability and feasibility, as it retained more than 91% of its degrading efficiency during five consecutive cycles, and showed remarkable activity in various water samples. This study emphasizes the utility and uniqueness of using environmentally friendly Ag/ZnO nanoparticles along with the use of biodegradable polymer support to produce a green material with antimicrobial and wastewater treatment properties in visible light.

Future work should therefore concentrate on overcoming existing limitations through investigating the performance of Ag/ZnO/CMC nanocomposite as a catalyst for the degradation of industrial wastewater that contains various types of contamination, instead of using artificial dye solutions. Moreover, thorough studies have to be conducted to reveal its environmental toxicity, cytotoxic effects, as well as metal ion leakage after prolonged use, in order to avoid negative consequences when applying this catalyst on an environmentally-biomedical scale. Additionally, radical scavenging methods and *in situ* spectroscopy should be applied for elucidation of photocatalytic processes, including participation of ROS and their role in the formation of intermediates. Further research is also required in scaling the synthesis process and improving regeneration and stability, along with refining the interfaces between the polymer matrix and semiconductors for better visible-light absorption capability and catalytic activity. In addition, expansion of its application to the degradation of antibiotics, inhibiting the formation of biofilms, and water purification from the sun are potential directions for future research that could increase the scope of its applications. Particularly, this work provides a promising green, cost-effective, and multifunctional nanoplatform that can contribute to sustainable wastewater treatment, antimicrobial protection, and future environmental remediation technologies worldwide.

## Conflicts of interest

The authors declare that they have no known competing financial interests or personal relationships that could have influenced the work reported in this paper.

## Supplementary Material

RA-OLF-D6RA04414J-s001

## Data Availability

The data supporting the findings of this study are available within the article and its supplementary information (SI). Supplementary information: characterization and performance data supporting the main fin–ings. Tables S1-S4 and Fig. S1 present UV-Vis, FTIR, EDX, elemental mapping, and TEM particle-size data. Tables S5 and S6 provide antibacterial activity and MIC results. Tables S7 and S8 summarize the effects of operational parameters and comparative CV photocatalytic degradation, respectively. Tables S9–S12 present kinetic and adsorption modeling results. Tables S13 and S14 report photocatalyst reusability and FTIR analysis of CV degradation, respectively, while Tables S15 and S16 provide Ag^+^/Zn^2+^ leaching and radical-scavenging results, respectively. See DOI: https://doi.org/10.1039/d6ra04414j.
