# Supplementary material for: Biogenic silver/zinc oxide-carboxymethyl cellulose nanocomposites with enhanced photocatalytic and antimicrobial activities for environmental remediation
Source: RSC Adv. 2026 Sep 15;16(44):55126–53. doi: 10.1039/d6ra04414j (PMC13575757; doi:10.1039/d6ra04414j)
Supplement: Supplementary file 1 [file RA-016-D6RA04414J-s001.pdf]

Supplementary Information (SI) for RSC Advances.
This journal is © The Royal Society of Chemistry 2026

Supplementary Materials

Biogenic Silver/Zinc Oxide-Carboxymethyl Cellulose Nanocomposites with Enhanced Photocatalytic and Antimicrobial Activities for Environmental Remediation

Section S1. Materials and Methods

1. Reagents and Chemicals

All chemicals and reagents used in this study were of analytical grade and were utilized without further purification unless otherwise stated. Distilled water was used throughout all experimental procedures. Silver nitrate ($AgNO_3$, ≥99.9% purity, Sigma-Aldrich, USA) was employed as the silver precursor. Zinc acetate dihydrate [$Zn(CH_3COO)_2 \cdot 2H_2O$, ≥99.0% purity, Merck, Germany] was used as the zinc source. Carboxymethyl cellulose (CMC, medium viscosity, and degree of substitution ca. 0.7, Sigma-Aldrich, USA) was used as the polymer matrix for nanocomposite formation. Crystal Violet dye ($C_{25}H_{30}ClN_3$, dye content ≥88%, Sigma-Aldrich, USA) was used as the model organic pollutant for photocatalytic degradation experiments. The materials utilized in the pH regulation experiments for photocatalysis were hydrochloric acid (HCl, 37%, Merck, Germany) and sodium hydroxide (NaOH, ≥98%, Sigma-Aldrich, USA). The effect of varying ionic strength on the process was evaluated using calcium chloride ($CaCl_2$, ≥99%, Merck, Germany). Ethanol (C_2H_5OH, ≥99.8%, Merck, Germany) was used to wash and purify the nanocomposites. Nutrient agar (HiMedia Laboratories, India) and nutrient broth (HiMedia Laboratories, India) were used for culturing bacteria. Microbiological ware and nutrient media used were sterilized before use. Fresh bark of *Cinnamomum verum* was obtained locally and authenticated before use. Plant extract served as a natural reducing, stabilizing, and capping agent in nanoparticle synthesis.

2. Instruments

UV-Vis spectroscopy analysis was run on Spekol 11, Analytik Jena AG, Jena, Germany. SEM and EDX analysis were conducted on a Czech FEI SEM-type instrument. FTIR analysis was employed on a Bruker spectrometer. X-ray diffraction (XRD) analysis was conducted on a Pan Analytical Philips. Sonication was performed using an Elma Schmidbauer GmbH (Gottlieb-Daimler-Straße 17, Singen, Germany) sonicator during nanoparticle preparation. Subsequently, a Beckman Coulter Allegra X-15R centrifuge (Beckman Coulter, Inc., California, USA) was employed during the synthesis of the nanoparticles. The spectrophotometric apparatus (Spekol 11 spectrophotometer, analytic Jena AG, Jena, Germany) was used for antioxidant analysis and dye degradation. BET analysis was performed on BELSORP MINI X Features

(version 7), while temperature-programmed desorption analysis was applied on BELCAT II. The CO_2 and NH_3 temperature-programmed desorption analysis was applied to investigate the fundamental surface properties of the nanocomposites. The process involved a series of gas flow and temperature-controlled steps programmed to establish the distribution and strength of basic sites. The sample was pre-treated for the first time under a helium (He) gas flow of 25 sccm and then heated to 400 °C for 30 minutes, further 45 minutes at the same temperature to ensure proper cleaning of the surface. The system was allowed to cool down to 50 °C under He for 1 minute using a cooling fan, followed by stabilization for 15 minutes at the same temperature without a fan. CO_2 adsorption was carried out by exposing the sample to CO_2 or NH_3 gas flow (25 sccm) at 50 °C for 60 minutes. Lastly, a He purge was carried out at 25 sccm for 15 minutes to desorb the loosely adsorbed CO_2 or NH_3 molecules. The desorption profile collected during the programmed heating stage provided detailed information regarding the surface basicity. The identified CO_2-TPD and NH_3-TPD peaks were attributed to weak, medium, and strong basic sites and unveiled the presence of a heterogeneous distribution of basic sites, which is important in enhancing the adsorption and catalytic character of the nanocomposite.

3. Broth Microdilution Assay for MIC Determination

The antibacterial activity of the Ag/ZnO/CMC nanocomposite (NC) was quantitatively evaluated using a broth microdilution assay [1] against *Salmonella typhimurium*, *Klebsiella pneumoniae*, *Bacillus subtilis*, and *Staphylococcus aureus*. Briefly, bacterial cultures were grown in appropriate sterile nutrient broth and adjusted to a standardized inoculum before use. Serial two-fold dilutions of the Ag/ZnO/CMC NC were prepared in sterile broth to obtain final concentrations of 0.781, 1.563, 3.125, 6.25, 12.5, 25, 50, and 100 µg mL^{-1}.

Aliquots of the standardized bacterial suspension were added to the wells containing the different NC concentrations and incubated under suitable growth conditions. Untreated bacterial cultures without NC were included as growth controls, while broth without bacterial inoculum was used as a sterility/background control. Following incubation, bacterial growth was assessed by measuring the optical density at 600 nm (OD_{600}) using a microplate reader. Each concentration was tested in three independent experiments, and the results were expressed as the mean OD_{600} value.

The minimum inhibitory concentration (MIC) was defined as the lowest concentration of Ag/ZnO/CMC NC that effectively inhibited bacterial growth, as indicated by the absence of a significant increase in OD600 compared with the corresponding untreated growth control. Based on the broth microdilution results, the MIC values were 12.5 µg mL^{-1} for *S. typhimurium*, 6.25 µg mL^{-1} for *K. pneumoniae*, 3.125 µg mL^{-1} for *B. subtilis*, and 6.25 µg mL^{-1} for *S. aureus*.

Section S2. Results

Table S1. UV-Vis absorption characteristics and optical band gap parameters of cinnamon extract, Ag/ZnO NC, and Ag/ZnO/CMC NC, including absorption maxima, absorbance values, and band gap energies estimated from Tauc plot analysis.

Sample	Absorbance	Wavelength (nm)
**(A) Cinnamon extract**	0.378	464.0
**(B) Ag/ZnO NC**	0.615	564.0
**(C) Ag/ZnO/CMC NC**	1.18	581.0

Table S2. FTIR spectral analysis of cinnamon extract, Ag/ZnO nanocomposite, and Ag/ZnO/CMC nanocomposite: assignment of functional groups and interpretation of their role in nanoparticle formation and stabilization.

Sample	Wavenumber (cm^{-1})	Tentative Functional Group	Interpretation
**Cinnamon Extract**	3376, 3227	-OH (phenols, alcohols)	Hydrogen-bonded hydroxyl groups of polyphenols are responsible for reducing and capping nanoparticles
	2927	-CH (alkanes)	Aliphatic C-H stretching of cinnamon biomolecules
	1607, 1575, 1522	C=C (aromatic), C=O (amide)	Aromatic ring vibrations and carbonyl groups contribute to nanoparticle stabilization
	1456, 1410, 1381	$-CH_2$ bending, $-COO^-$	Functional groups interacting with metal ions
	1258, 1202, 1151	C-O, C-N stretching	Phenolic and amide functionalities are involved in the reduction
	1055, 1020	C-O (alcohols, ethers)	Stabilizing interactions with the nanoparticle surface
	865, 771, 734	Aromatic =C-H bending	Phytochemical backbone vibrations
	627, 516, 489, 420	-	Minor fingerprint region peaks
**Ag/ZnO NC**	3217	-OH	Reduced intensity indicates partial involvement in metal reduction
	1773	C=O (ketone, aldehyde)	Interaction with the metal surface
	1609, 1512	C=C aromatic	Stabilization of nanoparticles
	1413, 1341	$-CH_2$, $-COO^-$	Metal-ligand interactions
	1269, 1223	C-O, C-N	Surface capping by biomolecules
	1116, 1050	C-O	Metal oxide formation indication
	883, 823, 770	Aromatic	Residual phytochemicals

		bending	
	647, 546, 507, 419	Ag-O, Zn-O	Confirmation of Ag/ZnO nanocomposite formation
**Ag/ZnO/CMC NC**	3358	-OH	Hydrogen-bonding with CMC, nanoparticle stabilization
	1615, 1602	C=O (amide, carboxyl)	Interaction with Ag/Zn surface, enhanced stability
	1435, 1339	$-CH_2$, $-COO^-$	CMC polymer matrix binding
	1251, 1151, 1108	C-O, C-N	Polymer encapsulation of a nanocomposite
	1021	C-O-C	Crosslinking within the CMC matrix
	823, 666, 555, 521, 395	Metal-O	Confirmation of Ag/ZnO within CMC

Table S3. The results of EDX analysis of Ag/ZnO and Ag/ZnO/CMC NCs.

Sample	Weight %	Atomic %	Error %
**Ag/ZnO NC**			
**O**	33.64	76.83	16.97
**Ag**	63.25	21.43	4.26
**Zn**	3.11	1.74	13.14
**Sample**	**Weight %**	**Atomic %**	**Error %**
**Ag/ZnO/CMC NC**			
**C**	0.60	1.37	91.71
**O**	48.64	83.91	11.71
**Ag**	40.38	10.33	3.42
**Zn**	10.38	4.39	6.30

Table S4. The results of elemental mapping analysis of Ag/ZnO and Ag/ZnO/CMC NCs.

Sample	Elements	% Composition
**Ag/ZnO NC**	O	8
	Ag	88
	Zn	4
**Ag/ZnO/CMC NC**	C	4
	O	17
	Ag	65
	Zn	14

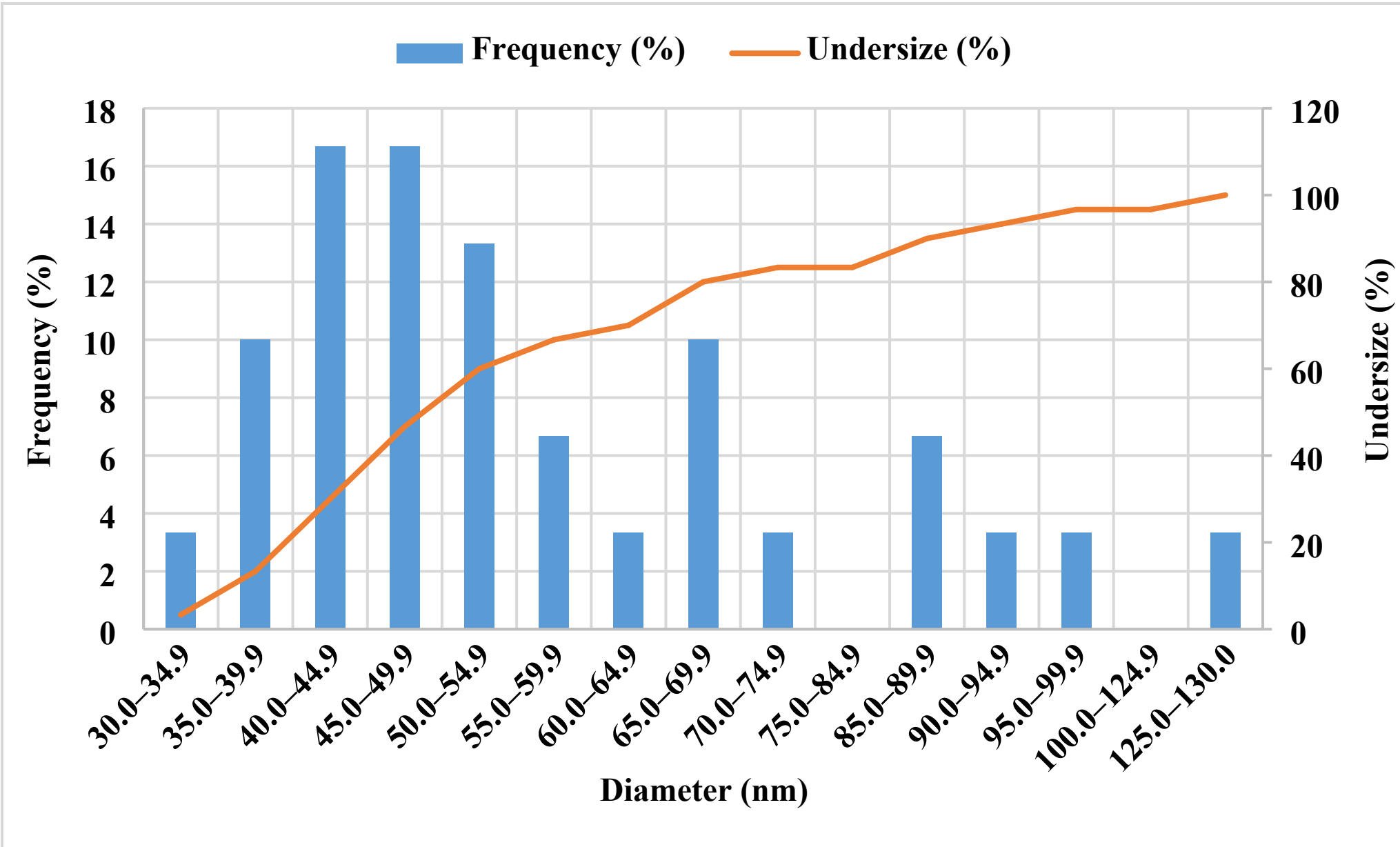

Figure S1. TEM-derived particle-size distribution of Ag/ZnO/CMC NC based on measurements of 30 individual nanoparticles from representative TEM micrographs.

Table S5. Antibacterial activity of cinnamon extract, Ag/ZnO NC, and Ag/ZnO/CMC NC against different bacterial strains expressed as inhibition zone diameters (mm). Data are presented as mean ± standard deviation (SD) of three independent experiments ($n = 3$). Statistical analysis was performed using one-way ANOVA followed by Tukey's post hoc test at $p < 0.05$. Different superscript letters within the same row indicate statistically significant differences among treatments.

Microorganisms	Cinnamon extract	Ag/ZnO NC	Ag/ZnO/CMC NC	Azithromycin
Gram-negative bacteria				
***Escherichia coli* (ATCC 10536)**	NA	22.0 ± 0.8^{b}	30.0 ± 1.0^{a}	18.0 ± 0.7^{c}
***Salmonella typhimurium* (ATCC 25566)**	NA	22.0 ± 0.6^{b}	32.0 ± 1.1^{a}	17.0 ± 0.5^{c}
***Klebsiella pneumoniae* (ATCC 10031)**	NA	21.0 ± 0.7^{b}	32.0 ± 0.9^{a}	18.0 ± 0.6^{c}
***Enterobacter cloacae* (DMS 30054)**	NA	21.0 ± 0.5^{b}	30.0 ± 0.8^{a}	NA
Gram-positive bacteria				
***Bacillus subtilis* (DMS 1088)**	NA	21.0 ± 0.6^{c}	33.0 ± 1.0^{a}	29.0 ± 0.9^{b}
***Bacillus cereus* (EMCC 1080)**	NA	21.0 ± 0.7^{c}	31.0 ± 1.1^{a}	20.0 ± 0.8^{c}
***Staphylococcus aureus* (ATCC 6538)**	NA	23.0 ± 0.8^{c}	36.0 ± 1.2^{a}	32.0 ± 1.0^{b}
***Staphylococcus epidermidis* (EMCC 1353T)**	NA	21.0 ± 0.6^{c}	32.0 ± 1.0^{a}	29.0 ± 0.9^{b}

"NA" indicates no detectable antibacterial activity.

Table S6. Optical density (OD_{600}) values obtained from the broth microdilution assay for Ag/ZnO/CMC NC against *S. typhimurium*, *K. pneumoniae*, *Bacillus subtilis*, and *S. aureus*. The minimum inhibitory concentration (MIC) was defined as the lowest nanocomposite concentration that prevented visible bacterial growth, as determined by comparison of OD_{600} values with the untreated growth control. Data are presented as the mean of three independent experiments.

Bacterial strain	Conc. (μg mL^{-1})	OD_{600}	Bacterial strain	Conc. (μg mL^{-1})	OD_{600}
S. typhimurium	**100**	0.01	***B. subtilis***	**100**	0.00
	**50**	0.02		**50**	0.01
	**25**	0.03		**25**	0.02
	**12.5**	0.03		**12.5**	0.02
	**6.25**	0.58		**6.25**	0.03
	**3.125**	0.69		**3.125**	0.05
	**1.563**	0.81		**1.563**	0.36
	**0.781**	0.95		**0.781**	0.52
**MIC**	**12.5**		**MIC**	**3.125**	
K. pneumoniae	**100**	0.01	***S. aureus***	**100**	0.00
	**50**	0.02		**50**	0.01
	**25**	0.02		**25**	0.02
	**12.5**	0.03		**12.5**	0.04
	**6.25**	0.05		**6.25**	0.05
	**3.125**	0.42		**3.125**	0.50
	**1.563**	0.56		**1.563**	0.72
	**0.781**	0.74		**0.781**	0.96
**MIC**	**6.25**		**MIC**	**6.25**	

Table S7. Photocatalytic degradation of Crystal Violet (CV) using Ag/ZnO/CMC NC (Mean ± SD, n = 3).

Parameter	Condition	Degradation (%)
**pH**	2	71.27 ± 2.10
	4	77.75 ± 2.00
	6	83.77 ± 1.80
	8	89.60 ± 1.60
	10	93.52 ± 1.50
**Time (min)**	15	47.58 ± 2.50
	30	67.38 ± 2.20
	60	87.45 ± 1.80
	120	92.17 ± 1.50
	180	99.33 ± 1.00
**Temperature (°C)**	25	91.00 ± 1.20
	35	88.29 ± 1.30
	45	78.86 ± 1.60
	55	65.34 ± 2.10
**NC concentration (mg/mL)**	0.10	72.73 ± 2.30
	0.25	76.04 ± 2.10
	0.50	86.82 ± 1.70
	0.75	91.36 ± 1.40
	1.00	99.45 ± 0.90
**Ionic strength ($CaCl_2$)**	0 µL	99.28 ± 0.90
	250 µL	91.50 ± 1.20
	500 µL	87.03 ± 1.40
	1250 µL	81.27 ± 1.70
	2500 µL	76.16 ± 2.10

Table S8. Comparative photocatalytic degradation of Crystal Violet (CV) under visible-light irradiation using CMC, Ag/CMC, ZnO/CMC, an Ag + ZnO/CMC physical mixture, and the Ag/ZnO/CMC nanocomposite. Values are presented as mean ± standard deviation (SD, n = 3). Blank represents CV solution without catalyst.

Catalyst	CV degradation (%)
**Blank**	5.8±0.74
**CMC**	10.6±1.18
**Ag/CMC**	74.8±1.41
**ZnO/CMC**	67.5±1.52
**Ag + ZnO/CMC physical mixture**	84.7±1.33
**Ag/ZnO/CMC NC**	99.33±1.01

Table S9. Pseudo-first-order kinetic model for Crystal Violet degradation using Ag/ZnO/CMC NC.

Time (min)	Degradation (%)	C_t/C_0	$\ln(C_0/C_t)$
**0**	0.00 ± 0.00	1.0000 ± 0.0000	0.000 ± 0.000
**15**	47.58 ± 2.50	0.5242 ± 0.0200	0.646 ± 0.021
**30**	67.38 ± 2.20	0.3262 ± 0.0180	1.120 ± 0.030
**60**	87.45 ± 1.80	0.1255 ± 0.0110	2.076 ± 0.042
**120**	92.17 ± 1.50	0.0783 ± 0.0080	2.549 ± 0.051
**180**	99.33 ± 1.00	0.0067 ± 0.0010	5.010 ± 0.070
**Parameter**	**Value**		
**Rate constant (k, min^{-1})**	0.0251		
**Regression equation**	$\ln(C_0/C_t) = 0.0251t + 0.2091$		
**R^2**	0.9539		
**Half-life ($t_{1/2}$) (min)**	24.2		
**Kinetic model**	Pseudo-first-order		

Table S10. Langmuir adsorption isotherm parameters for Crystal Violet adsorption on Ag/ZnO/CMC NC.

Ce (mg/L)	qe (mg/g)	Ce/qe
**10 ± 0.30**	8.10 ± 0.25	1.235 ± 0.030
**20 ± 0.40**	15.80 ± 0.35	1.266 ± 0.028
**30 ± 0.50**	23.50 ± 0.42	1.277 ± 0.026
**40 ± 0.60**	31.00 ± 0.48	1.290 ± 0.025
**50 ± 0.70**	38.20 ± 0.55	1.309 ± 0.024
**Parameter**	**Value**	
**Qmax (mg/g)**	62.85 ± 1.42	
**KL (L/mg)**	0.082 ± 0.004	
**R^2**	0.9652	
**Adsorption behavior**	Favorable monolayer adsorption	

Table S11. Freundlich adsorption isotherm parameters for Crystal Violet adsorption on Ag/ZnO/CMC NC.

Ce (mg/L)	qe (mg/g)	log Ce	log qe
**10 ± 0.30**	8.10 ± 0.25	1.000 ± 0.010	0.908 ± 0.012
**20 ± 0.40**	15.80 ± 0.35	1.301 ± 0.012	1.199 ± 0.015
**30 ± 0.50**	23.50 ± 0.42	1.477 ± 0.014	1.371 ± 0.017
**40 ± 0.60**	31.00 ± 0.48	1.602 ± 0.015	1.491 ± 0.019
**50 ± 0.70**	38.20 ± 0.55	1.699 ± 0.016	1.582 ± 0.020
**Parameter**	**Value**		
**KF**	5.74 ± 0.18		
**n**	2.41 ± 0.09		
**R^2**	1.0		
**Adsorption behavior**	Favorable heterogeneous adsorption		

Table S12. Langmuir-Hinshelwood kinetic model parameters for Crystal Violet degradation using Ag/ZnO/CMC NC.

Time (min)	Degradation (%)	Ct/C₀	ln(C₀/Ct)
**0**	0.00 ± 0.00	1.0000 ± 0.0000	0.000 ± 0.000
**15**	47.58 ± 2.50	0.5242 ± 0.0200	0.646 ± 0.021
**30**	67.38 ± 2.20	0.3262 ± 0.0180	1.120 ± 0.030
**60**	87.45 ± 1.80	0.1255 ± 0.0110	2.076 ± 0.042
**120**	92.17 ± 1.50	0.0783 ± 0.0080	2.549 ± 0.051
**180**	99.33 ± 1.00	0.0067 ± 0.0010	5.010 ± 0.070

Parameter	Value
**Kinetic model**	Langmuir-Hinshelwood (pseudo-first-order form)
**Apparent rate constant (kapp) (min^{-1})**	0.0286 ± 0.0012
**Adsorption constant (K_{ads}) (L/mg)**	0.082 ± 0.004
**Regression equation**	$\ln(C_0/Ct) = 0.0251t + 0.2091$
**R^2**	0.9539
**Half-life ($t_{1/2}$) (min)**	24.2
**Model validity**	High

Table S13. Reusability and stability performance of Ag/ZnO/CMC NC during repeated photocatalytic degradation cycles of Crystal Violet under visible light irradiation. Data are presented as mean ± SD (n = 3).

Cycle Number	Degradation Efficiency (%)	Residual Activity (%)	Observation
**1**	99.33 ± 1.00	100.0	Fresh catalyst with maximum activity
**2**	97.84 ± 1.12	98.5	Negligible loss in activity
**3**	95.92 ± 1.25	96.6	Stable photocatalytic performance
**4**	93.76 ± 1.38	94.4	Slight decrease due to surface fouling
**5**	91.48 ± 1.52	92.1	Good recyclability retained

Table S14. FTIR band assignments and interpretation of Crystal Violet (CV) dye before and after treatment with Ag/ZnO/CMC nanocomposite (NC), demonstrating structural degradation and chromophore destruction during the photocatalytic process.

Sample	Wavenumber (cm^{-1})	Functional Group Assignment	Interpretation
**Crystal Violet**	3380, 3190	O-H and N-H stretching	Adsorbed water molecules and amine groups
**Crystal Violet**	1660	C=N stretching	Chromophoric structure responsible for violet coloration
**Crystal Violet**	1580, 1520	Aromatic C=C stretching	The presence of benzene rings in triphenylmethane structure
**Crystal Violet**	1480-1290	C-N stretching and aromatic skeletal vibration	Dimethylamino groups and aromatic framework
**Crystal Violet**	1220, 1160, 1110, 1060	C-N and C-C stretching	Structural vibrations of aromatic amine system
**Crystal Violet**	<1000	Aromatic C-H bending	Substituted benzene ring deformation
**CV treated with Ag/ZnO/CMC NC**	3350	O-H stretching	Formation of hydroxylated intermediates and adsorbed water
**CV treated with Ag/ZnO/CMC NC**	Disappearance of the band at 1660	Loss of C=N chromophore	Oxidative degradation and destruction of dye chromophore
**CV treated with Ag/ZnO/CMC NC**	Reduced 1520-1220 region	Decrease in aromatic skeletal vibrations	Breakdown of triphenylmethane aromatic structure
**CV treated with Ag/ZnO/CMC NC**	1580, 1420	Residual aromatic vibrations	Formation of partially oxidized intermediates
**CV treated with Ag/ZnO/CMC NC**	1020, 990	Oxygenated functional groups	Formation of low-molecular-weight oxidation products
**CV treated with Ag/ZnO/CMC NC**	Simplified FTIR spectrum	Loss of characteristic dye bands	Evidence of photocatalytic degradation and partial mineralization

Table S15. Ag^+ and Zn^{2+} leaching from Ag/ZnO/CMC NC at different catalyst concentrations.

Ag/ZnO/CMC NC concentration (g L^{-1})	Ag^+ leaching (mg L^{-1})	Zn^{2+} leaching (mg L^{-1})	Ag released (%)	Zn released (%)
**0.25**	0.08 ± 0.01	0.21 ± 0.02	0.16 ± 0.02	0.42 ± 0.04
**0.50**	0.15 ± 0.02	0.39 ± 0.03	0.15 ± 0.02	0.39 ± 0.03
**0.75**	0.22 ± 0.08	0.56 ± 0.04	0.15 ± 0.01	0.37 ± 0.03
**1.00**	0.29 ± 0.03	0.72 ± 0.05	0.15 ± 0.02	0.36 ± 0.03
**1.50**	0.41 ± 0.07	1.02 ± 0.07	0.14 ± 0.01	0.34 ± 0.02
**2.00**	0.53 ± 0.11	1.29 ± 0.09	0.13 ± 0.01	0.32 ± 0.02

Table S16. Effect of selective radical scavengers on the photocatalytic degradation of Crystal Violet by Ag/ZnO/CMC NC.

Scavenger	Target reactive species	Scavenger concentration	CV degradation (%)	Inhibition (%)
**None**	-	-	99.33 ± 1.00	-
**Isopropanol (IPA)**	$^\bullet OH$	10 mM	58.42 ± 2.15	41.20
***p*-Benzoquinone (BQ)**	$O_2^{\bullet -}$	1 mM	69.76 ± 1.84	29.84
**EDTA-2Na**	h^+	1 mM	82.15 ± 1.63	17.28
**Sodium azide (NaN_3)**	$^1O_2$	1 mM	91.47 ± 1.21	7.91

References

1. Schön, T., Werngren, J., Machado, D., Borroni, E., Wijkander, M., Lina, G., Mouton, J., Matuschek, E., Kahlmeter, G., Giske, C. and Santin, M., 2020. Antimicrobial susceptibility testing of *Mycobacterium tuberculosis* complex isolates-the EUCAST broth microdilution reference method for MIC determination. Clinical Microbiology and Infection, 26(11), pp.1488-1492.
